# Molecular and morphological data suggest a new species of big-eared bat (Vespertilionidae: *Corynorhinus*) endemic to northeastern Mexico

**DOI:** 10.1371/journal.pone.0296275

**Published:** 2024-02-21

**Authors:** Issachar L. López-Cuamatzi, Jorge Ortega, Sandra M. Ospina-Garcés, Gerardo Zúñiga, M. Cristina MacSwiney G.

**Affiliations:** 1 Centro de Investigaciones Tropicales, Universidad Veracruzana, Xalapa de Enríquez, Veracruz, Mexico; 2 Departamento de Zoología, Instituto Politécnico Nacional, Escuela Nacional de Ciencias Biológicas, Mexico City, Mexico; 3 Centro de Investigación en Biodiversidad y Conservación, Universidad Autónoma del Estado de Morelos, Cuernavaca, Morelos, Mexic; University of Veterinary Medicine Vienna: Veterinarmedizinische Universitat Wien, AUSTRIA

## Abstract

*Corynorhinus mexicanus* is an insectivorous bat endemic to Mexico that inhabits the high and humid regions of the Sierra Madre Oriental (SMO), the Trans-Mexican Volcanic Belt (TMVB), and the Sierra Madre Occidental (SMOC). A previous study suggested that *C*. *mexicanus* could be a cryptic species complex due to the genetic divergence observed between specimens from the TMVB and SMOC. The present study implemented phylogenetic, population genetics, and morphological analyses to evaluate the hypothesis that *C*. *mexicanus* is a species complex. The phylogenetic analysis indicated that *C*. *mexicanus* is a polyphyletic species composed of three indirectly related lineages. The estimated divergence times for the lineages suggest that they first originated during the Pliocene, while the second and third shared a common ancestor with *C*. *townsendii* 1.55 million years ago, and diverged 600,000 years ago during the Middle Pleistocene. The population genetics analysis reveals the SMO lineage of *C*. *mexicanus* is an isolated genetic group and highly diverged from the rest of lineages (SMOC and TMVB). The morphological analyses showed variation in the skull and mandible associated with the lineages and sex of the specimens, highlighting a difference in mandible shape between the specimens of the SMO and the rest of *C*. *mexicanus*. The results of this study suggest the presence of an undescribed species of the genus *Corynorhinus*.

## Introduction

Bats are one of the most diverse and also one of the most threatened mammal groups [1]. Certain basic aspects of the biology of bats remain unknown, hindering the development of conservation strategies [1]. One such aspect is related to the taxonomy and systematics of species since cryptic species complexes are common in bats [2]. Conservation efforts may be misdirected because they are focused on a “single” species when there are two or more putative species with different evolutionary histories and conservation demands. Systematics can therefore operate together to better define the species that conservation programs seek to protect [3–5].

Cryptic species are common in many taxa, including bats [2]. Possible reasons for this are suggested by Fiser et al. [6]: i) recent divergence (e.g., Mustached bats, genus *Pteronotus*; [7]), ii) phylogenetic niche conservatism or morphological stasis (e.g., Mastiff bats, genus *Molossus*; [8]) and iii) morphological convergence (e.g., Hoary bats, genus *Lasiurus*; [9]). In addition, despite their ability to fly, not all bat species can disperse over large distances [10]. Genetic interchange in a population is therefore conditional on geographical proximity, with populations that inhabit isolated lands (such as islands or mountains) being more susceptible to genetic divergence and speciation [11].

With the arrival of the molecular and genomic era, most new bat species described in recent decades have been a product of resolved cryptic species complexes [12–16]. However, the unique use of molecular data for splitting species has caused some debate since not all molecular markers are useful for disentangling species [3]. In response to the reductionist approach of the exclusive use of molecular data in species delimitation, a holistic conception has resurfaced. This conception, known as integrative taxonomy, seeks to establish limits between species through the criteria of accumulation and congruence of different lines of evidence (molecular, ecological, behavioral, acoustic, and/or morphological) that support the persistence of a taxonomic entity [3,17,18].

The discovery and disentangling of cryptic species complexes using an integrative approach has resolved the taxonomy of the bat species that inhabit Mexico (i.e., *Glossophaga mutica*, [19]; *Chiroderma scopaeum*, [20]; *Lophostoma nicaraguae*, [21]; *Pteronotus mesoamericanus*, *P*. *mexicanus*, *P*. *psilotis*, *P*. *fulvus* [22]), and highlights the need for re-evaluation of those potential cryptic species, especially those included in a risk category of the IUCN-Red List or protected by local or regional conservation laws.

The genus *Corynorhinus* (Allen, 1965) [23], also known as North American big-eared bats, includes three species [24]: *C*. *rafinesquii* (Lesson, 1827) [25], *C*. *townsendii* (Cooper, 1838) [26], and *C*. *mexicanus* Allen, 1916 [27]. According to Medellín [28] and Ramírez-Pulido et al. [29] there are two species of the genus *Corynorhinus* recognized in Mexico: Townsend’s big-eared bat (*C*. *townsendii*), which occurs from Baja California and Sonora through the Mexican Plateau to southern Mexico in Chiapas, and the Mexican big-eared bat (*C*. *mexicanus*), which is an endemic species listed as Near Threatened by the IUCN [30] and distributed in the Sierra Madre Occidental (SMOC), Sierra Madre Oriental (SMO) and Trans-Mexican Volcanic Belt (TMVB) [30,31]. Some records of *C*. *mexicanus* exist from the Yucatan Peninsula, but these are considered unreliable [32] since they are out of the range of distribution. Moreover, these records occurred in tropical lowland deciduous forests, whereas *C*. *mexicanus* is normally found in temperate and humid pine-oak forests associated with mountain systems. Piaggio and Perkins [33] provided the most recent phylogenetic revision of the genus and support the taxonomy arrangement of the three species previously proposed [24]. However, these authors suggest the possibility of a cryptic species complex within *C*. *mexicanus* [33]. This hypothesis was based on the high intraspecific genetic distance found in individuals from Mexico City (located on the TMVB) and Durango (located in the SMOC) [33]. However, since the sample size only included four individuals from two localities, the cryptic species hypothesis could not be corroborated [33]. On the other hand, studies of wing morphology [34] and foraging behavior [31,35,36] suggest that *C*. *mexicanus* presents low vagility [37,38], as is true for other *Corynorhinus* species [39]. Moreover, based on their specific roosting requirements, this species may have high fidelity to their roosting sites [40], which could affect inter-population connectivity and promote divergence and speciation within the Mexican big-eared bat.

Taking into consideration the ecological and morphological traits of *C*. *mexicanus* and the high interpopulation genetic distance founded by Piaggio and Perkins [33], we hypothesized *C*. *mexicanus* has the potential to be a cryptic species complex. In this study, we used an integrative approach to test whether *C*. *mexicanus* is a species complex. For this, we used mitochondrial and nuclear data to perform population genetic analysis and phylogenetic inference of geographically representative samples of this taxon to identify candidate lineages as putative species. We also conducted traditional and geometric morphometric analyses to detect morphological differences that support these putative species. Finally, we used the genetic [41,42] and phylogenetic species concept [43] and the accumulation and congruence criterion [18] as a baseline for delimiting taxa within *C*. *mexicanus*.

## Material and methods

### Genetic analysis

#### Sample collection

Fifty-five tissue samples of *C*. *mexicanus* were obtained from individuals captured in the field and from specimens preserved in scientific collections. In the field, tissue samples were obtained using a biopsy punch (3 mm in diameter) and making one incision by wing. We collected tissue from adults and subadult individuals of both sexes; only pregnant females or females with offspring attached to their bodies were released immediately in order to avoid stress on them. The tissue samples represented 15 geographical localities within the distribution range of *C*. *mexicanus* (Table A of S1 Appendix). Eleven additional tissue samples of *C*. *townsendii* were obtained in the field and from specimens preserved in scientific collections. These samples represent seven geographical localities and were used as an outgroup in the phylogenetic analysis and to guarantee accurate identification of the two species when both were caught in sympatry. We additionally collected as vouchers two non-reproductive adult females of *C*. *mexicanus* from Puerto Grande, Galeana, Nuevo León; and another one from La Malinche National Park, Tlaxcala. Voucher individuals were euthanized by placing them in a sealed chamber containing a cotton pad soaked with 5 ml of isoflurane. We used isoflurane, a volatile anesthetic that causes no specific signs of distress or pain in individuals [44], in strict accordance with the recommendations in the Guidelines of the American Society of Mammalogists for the use of wild mammals in research [45]. Cranium, skin, and tissues of voucher individuals from Nuevo León were deposited at Colección de Mamíferos, Museo de Zoología Alfonso L. Herrera, Universidad Nacional Autónoma de México (#catalog: MZFC-M16325 and MZFC-M16326), whereas cranium and skin of the individual from La Malinche, Tlaxcala, was deposited at Colección de Mamíferos, Instituto de Investigaciones Biologicas, Universidad Veracruzana (#catalog: IIB4365). Tissue samples of this individual were also deposited at Colección de Tejidos de Vertebrados de la Escuela Nacional de Ciencias Biológicas, Instituto Politécnico Nacional (#catalog: ENCB_Chis-Ves_0041). The field work and sample collection were carried out under Field Research Permit Number: SGPNDGVS/00365/22 granted by Secretaría del Medio Ambiente, Mexico.

#### DNA extraction, amplification, and sequencing

Total genomic DNA was extracted from tissue samples using the gDNA isolation kit ReliaPrep™ gDNA Tissue Miniprep System-PROMEGA®, following the manufacturer’s protocol, but resuspended in molecular grade water until a final volume of 100–200 μL. Partial sequences from the cytochrome c oxidase subunit I (COI) and cytochrome b (Cyt-*b*) mitochondrial genes and the recombination activating gene 2 (RAG2) nuclear gene were amplified using specific primers (Table A of S2 Appendix). PCR amplifications were performed in a Labnet MultiGene ™ Gradient PCR Thermal Cycler in a 25 mL final volume containing 2 μl of template DNA (50–200 ng/μl), 1 μl of each primer (10mM), 15 μl of Master Mix RED (AMPLIQON®, Denmark), and 6 μl of PCR-grade water. The amplification protocols varied among the molecular markers (Table B of S2 Appendix). DNA with no template was included in every round of PCR as a negative control to check for contamination. Amplicons were sequenced using both forward and reverse PCR primers in Macrogen, Inc. ® (Korea). DNA sequence data were edited in SEQUENCHER® and aligned in Clustal W implemented in MEGA X [46]. For RAG2 sequences, we used the International Union of Pure and Applied Chemistry (IUPAC) to code ambiguous variable nucleotide positions.

#### Population analysis

Haplotypes present in Cyt-*b*, COI, and Cyt-*b*+COI were obtained with DnaSP v. 6 [47]. Networks of these haplotypes were generated in POPART v. 1.7. [48] using the Median Joining algorithm [49]. The RAG2 haplotype network was obtained using a Bayesian approach implemented in PHASE v. 2.1 [50,51], which discards the presence of heterozygotes in sequences selecting haplotype pairs with a posterior probability of >0.90. To test the neutrality of the molecular markers, Tajima’s D and Fs’Fu metrics were performed in DnaSP v. 6 [47].

To infer spatial genetic discontinuities between *C*. *mexicanus* populations, individuals and concatenated genes were analyzed in GENELAND v. 4.9.2 [52] and STRUCTURE v. 2.3.4 [53]. We tested from k = 1 to k = 10 subdivisions in GENELAND, where ten independent runs were performed for each condition, with 1 million generations and a thinning of 100, using a true spatial model, uncorrelated genetic frequencies, and a value of 0.27° as a coordinate uncertainty that corresponds to the largest distance reported by *C*. *townsendii* [39]. Convergence of the results was reached after a burn-in of 1000, and the run with the highest likelihood value was selected. To detect population structure with STRUCTURE, we tested from k = 1 to k = 10 with 10 replicates each per condition. Population assignment was performed under an admixed and LocPrior model [54], with 1 million generations and a thinning of 1000. The results were evaluated using the Evanno method [55], as implemented in the Structure Harvester website [56].

Alternatively, we used analysis of molecular variance (AMOVA) to contrast the differentiation hypotheses of groups founded with STRUCTURE and GENELAND using Arlequin v. 3.5.2.2 [57,58]. Finally, to compare the genetic distances found among and within *C*. *mexicanus* populations, and among these and *C*. *townsendii*, pairwise genetic distances were estimated for Cyt-*b* with Kimura-2P model using MEGA X [46,59] after 1000 bootstrap pseudoreplicates.

#### Phylogenetic analysis

To assess whether nuclear and mitochondrial genes could be concatenated for the phylogenetic analysis, an incongruence analysis was performed in the MLSTests software v. 1.0.1.23 [60], using the BIONJ-ILD test, after 1000 pseudoreplicates. This method is a variant of the Incongruence Length Difference (ILD) test since it uses the BIO-neighbor Joining method instead of parsimony. We used the localized incongruence length difference test based on the neighbor joining method (NJ-LILD) to identify branch incongruence in the concatenated sequence tree. The statistical significance of the incongruence was evaluated with a modified Templeton test, which indicates whether the phylogenetic signals of topologically incompatible loci are well supported statistically.

The Templeton test indicated that RAG2 was causing topology incongruence (see the results section), and we therefore decided to run a phylogenetic analysis using nuclear and mitochondrial concatenated data separately. We conducted a phylogenetic analysis by Bayesian Inference (BI) and Maximum Likelihood (ML) using BEAST2 [61] and IQTREE2 [62], respectively. In both analyses, the sequences of *Plecotus auritus*, *Corynorhinus rafinesquii*, and *C*. *townsendii* were used as outgroups (#GenBank: DQ120821.1; GU328055.1; AY141029.1; AB085734.1; MT407322.1; NC_016872.1). We conducted a phylogenetic analysis using two datasets with different partition schemes. The first set was referred to as “*Codon position*”, and consisted of six partitions that represented the codon position of each gene (Table 1). For the second set, hereafter referred to as “*Best scheme*”, we *a priori* declared each codon position per gene (like the first set) and constructed combinations of these. *Best scheme* was then inferred using these combinations, the Bayesian Information Criterion (BIC), and the PartitionFinder [63] algorithm provided on the IQTREE2 platform. For ML, nucleotide substitution models were estimated for the partitions of each combination described above and using the substitution models available in IQTREE2 [64] (Table 1).

**Table 1:** Partition schemes of the Cyt-*b+*COI data and nucleotide substitution models used in the phylogenetic analysis.

Method of inference	Scheme	Partition	Model
Likelihood	Best scheme	1^st^ Cytb, 3^rd^ COI	HKY+Γ
		2^nd^ Cytb, 1^st^ COI	HKY+I
		3^rd^ Cytb, 2^nd^ COI	TIM+ Γ
	Codon position	1^st^ Cyt-*b*	HKY+Γ
		2^nd^ Cyt-*b*	HKY+I
		3^rd^ Cyt-*b*	TN+I
		1^st^ COI	F81
		2^nd^ COI	TIM+ Γ
		3^rd^ COI	K2P+I
Bayesian	Best scheme	1^st^ Cytb, 3^rd^ COI	HKY+Γ
		2^nd^ Cytb, 1^st^ COI	HKY+I
		3^rd^ Cytb, 2^nd^ COI	TN+Γ
	Codon position	1^st^ Cyt-*b*	HKY+Γ
		2^nd^ Cyt-*b*	HKY+I
		3^rd^ Cyt-*b*	TN+I
		1^st^ COI	HKY
		2^nd^ COI	TN+ Γ
		3^rd^ COI	HKY+I

The ML-phylogenetic analysis was performed in IQTREE2, with 10000 pseudoreplicates using the Ultrafast bootstrap (UFBoot, [65]). The BI-phylogenetic analysis was run in BEAST2 using the MCMC algorithm. Given that IQTREE2 and BEAST2 do not share the same nucleotide substitution models, in IQTREE2, we used the models available in BEAST2 (JC69, HKY85, TN93, and GTR). The best model was selected using the BIC criterion [61] (Table 1). The Bayesian analysis consisted of four independent chains, each of 20 million generations, sampling trees every 1000 generations, and using a burn-in of 10%. The convergence of results and good sampling (ESS > 200) was visualized in Tracer v. 1.7.2 [66]. All runs were combined in LogCombiner v. 2.6.4, and the final topology was obtained using a 0.5 posterior probability limit and a burn-in of 10%. The tree with maximum likelihood obtained in the ML analysis and the maximum clade credibility tree from BI were both visualized using FigTree v. 1.4.4 [67].

#### Multi-locus species tree analysis

A multi-locus species tree, including the RAG2, COI, and Cyt-*b* genes, was built with *BEAST [61]. We assigned the names of terminal taxa using the identities of genetic groups suggested by haplotype networks and genetic structure analyses. Sequences of *Plecotus auritus*, *Corynorhinus rafinesquii*, and *C*. *townsendii* were used as outgroups. The settings of the multispecies coalescent model were: i) a linear function with constant root as population, ii) the population means estimated by *BEAST, iii) strict molecular clock, iv) Yule model as the speciation model, and v) uniform *priors*. Nucleotide substitution models were the same as in the *Codon Position* scheme (Table 1) used for ML and BI phylogenetic analysis. Four chains were run in *BEAST, each consisting of 50 million generations, with sampling trees every 1000 generations and a burn-in of 10%. The convergence of results and good sampling (ESS > 200) were visualized in Tracer [66], all runs were combined in LogCombiner v. 2.6.4, and the final topology was obtained using a 0.5 posterior probability limit and a burn-in of 10%. The species tree was visualized using FigTree v. 1.4.4 [67] and Densitree v. 2.6.4. [68].

#### Mitochondrial phylogenomics

The presence of the molecular lineages was confirmed through a phylogenetic analysis of the genus *Corynorhinus*, using the entire mitochondrial genome (mitogenome). For this, the mitogenome of individual representatives of these lineages was sequenced using Illumina next-generation sequencing (S3 Appendix). The first individual of *C*. *mexicanus* came from La Malinche National Park (located in the TMVB) and was used as a representative of the SMOC and TMVB lineages. This decision was based on the low degree of genetic differentiation and phylogenetic relationship observed among those lineages (see the results section). The second individual came from Puerto Grande, located in Galeana, Nuevo León (SMO).

Sequences of the mitogenomes of *C*. *townsendii* (#GenBank CM047939.1) and *C*. *rafinesquii* (NC016872.1) were obtained from GenBank, as well as *Plecotus auritus* (HM164052.1), which was used as outgroup for phylogenetic analysis. Mitogenomes were aligned using the Clustal W algorithm implement in MEGA X, and gene annotation was established following annotation of the mitogenome of *Plecotus auritus* available in GenBank. Following Camacho et al. [69], the gene *nad6* and control region were excluded from the data matrix (see [70] for more details). The final data matrix consisted of five mitogenomes, each comprising 36 loci (two rRNA, 12 protein-coding, and 22 tRNA genes) and averaging 15 kb. We established a partition scheme following Camacho et al. [69]. This scheme consisted of 38 partitions: one for both ribosomal RNAs (12S and 16S), one for all transfer RNAs, and three for each protein-coding gene that represented the 1^st^, 2^nd^, and 3^rd^ codon position. For phylogenetic inference, selection of the best substitution model of each partition and determination of BI’s priors were conducted with the same criteria described in the section of phylogenetic analyses (Table A of S3 Appendix).

#### Estimation of divergence times

Divergence times among *C*. *mexicanus* lineages were estimated using only Cyt-*b* sequences since this gene was the most informative (see the results section). A single sequence per sampled locality was used in this analysis. Three preliminary analyses were run to check for any bias that may occur due to sequence selection (Table C and Fig A of S2 Appendix). Each analysis consisted of 10 million generations, with sampling of trees every 1000 generations and a burn-in of 10%. Sequences of *Plecotus auritus*, *Corynorhinus rafinesquii*, and *C*. *townsendii* were used to calibrate the tree height using previously published data [71]: divergence between *Plecotus-Corynorhinus* (6.585 ± 0.387 million years ago [Ma]) and origin of the *Corynorhinus* crown group (5.09 ± 0.86 Ma). As tree priors, we used a normal distribution prior for both calibrated points, the models HKY+ Γ for the 1^st^ and 2^nd^ codon positions, TN93+I for the 3^rd^ position, a relaxed lognormal molecular clock, and the Yule speciation model. Finally, the analysis consisted of four independent chains with 10 million generations, with sampling of trees every 1000 generations and a burn-in of 10%. The convergence of results and effective sampling (ESS > 200) was visualized in Tracer. The runs were combined in LogCombiner, and the final topology was obtained using a 0.5 posterior probability limit and a burn-in of 10%. The species tree was visualized using FigTree v. 1.4.4 [67].

### Morphometric analysis

#### Sample collection

A total of 158 *C*. *mexicanus* specimens were photographed and measured from nine mammal collections (Table B of S1 Appendix). Samples containing individuals of both sexes preserved in alcohol or dry, as well as those captured in the field from the SMOC, SMO, and TMVB, were used in traditional and geometric morphometric analyses.

#### External morphometric analysis

We used a digital caliper (Mitu-toyo CD-6´´ Mitutoyo U.S.A) with a precision of ± 0.1 mm to measure the lengths of the tibia, tragus, ear, and forearms. Additionally, we quantified the number of ridges on the uropatagium, also referred as interfemoral ridges. We count the ridges on both sides of the uropatagium, using the tail as the axis. This approach was adopted to account for instances where a singular ridge on the left side might bifurcate on the right side, or vice versa. Tumlison [72] reported sexual dimorphism in *C*. *mexicanus*, and differences among sexes were therefore evaluated using the t-test for tibia, tragus, ear, and forearm length, and Mann-Whitney’s U test for the interfemoral ridges. A two-way ANOVA was performed to determine whether sexual dimorphism varied within lineages found in the phylogenetic analysis, with sex nesting inside lineage in the model. Additionally, each morphological character was compared using one-way ANOVA among lineages, but with separation of the sexes. Only the number of interfemoral ridges were analyzed using a Kruskal-Wallis one-way analysis. Paired comparisons were conducted using the Tukey-Kramer *post hoc* method for unbalanced designs, with an error rate of 0.05 [73]. We also used Cohen’s D index as a measurement of effect size in all comparisons [74]. This index can be interpreted as follows: a small effect when D ≤ 0.2, a medium effect when 0.2 < D < 0.8, and a large effect when D ≥ 0.8 [74]. All ANOVAs were performed using the type III error recommended for unbalanced designs [75] and, for all parametric tests including ANOVAs, residual assumptions were evaluated using Shapiro-Wilk and Levene tests for normality and homoscedasticity, respectively, in the software JASP 0.16.3 [76].

#### Geometric morphometric analysis

**Fig 1:**
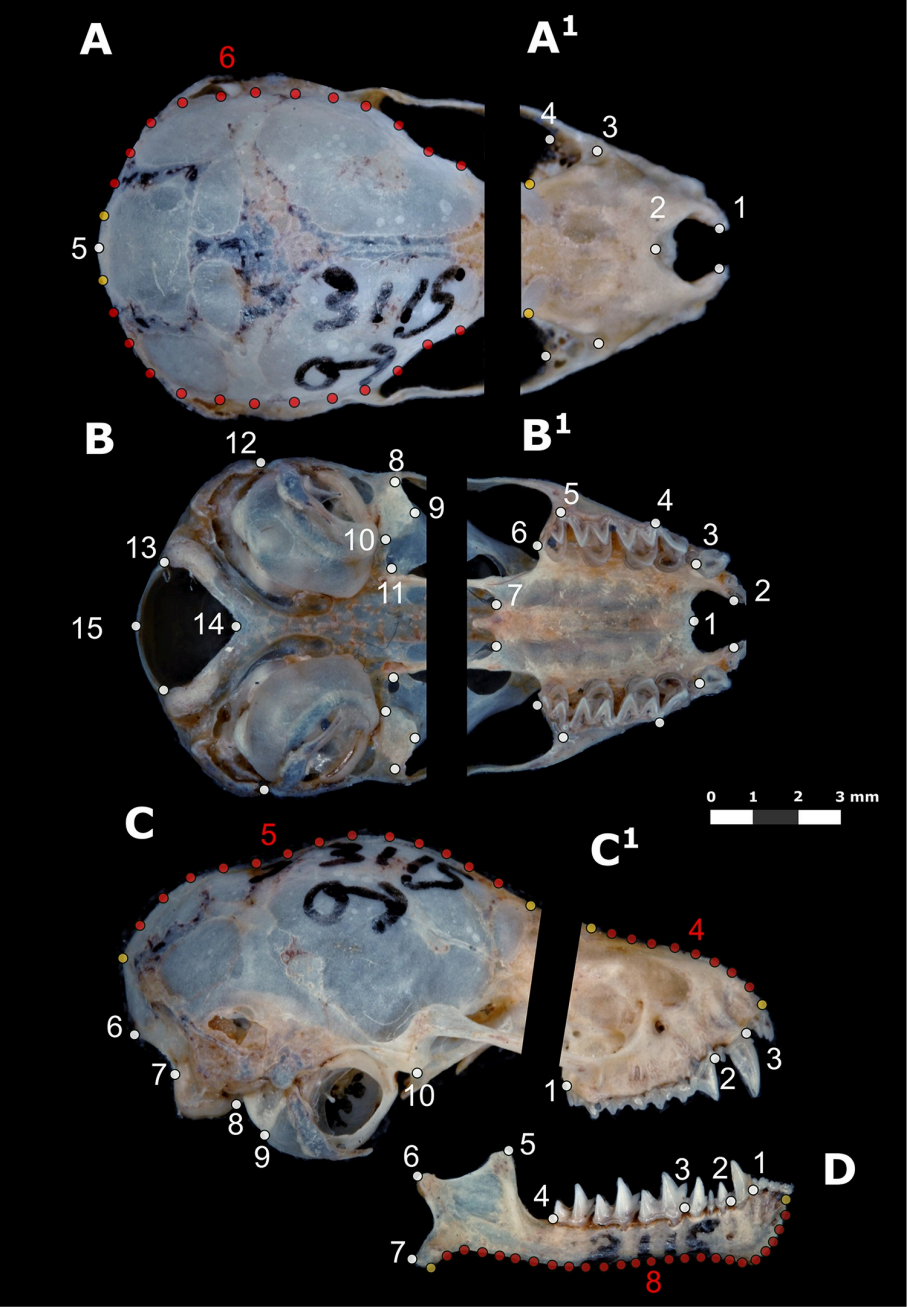
Views and modules of the skull and mandible of *Corynorhinus mexicanus* used in the morphometric analyses. **(**A), (B), and (C) correspond to the dorsal, ventral and lateral view of the basicranial module, respectively. (A^1^), (B^1^), and (C^1^) correspond to the same views of the rostral module. The lateral view of the mandible is shown in (D). The landmark configurations used for each view of the skull and mandible are shown with dots; yellow and white dots correspond to landmarks and red dots to semi-landmarks. Numbers indicate the identity of each mark (more details in S4 Appendix).

For geometric morphometric analysis, digital photographs of lateral, dorsal, and ventral views of skulls, and lateral views of mandibles, were obtained using a Nikon D3000 reflex camera (Nikon Corporation, Tokyo, Japan) with a Nikkor 2.8F 60 mm macro lens (Nikon Corporation, Tokyo, Japan). Photographs were taken while always maintaining the skulls and mandibles in the same position and at the same distance from the camera lens. Two-dimensional landmark and semi-landmark configurations were digitized on the skull and mandible digital images using the software tpsUtil and tpsDig2 [77,78]. All views of the skulls were divided into two modules, rostral and basicranial, which correspond to independent development modules supported for all mammals, including bats [79,80]. The number of landmarks and semi-landmarks used to describe the shape of the skull and mandible varied between modules and views (Fig 1, and S4 Appendix). Given the lack of homologous points in the central region of the mandible, it was analyzed as a whole using a geometric configuration of eight landmarks and 25 semi-landmarks (Fig 1).

Each landmark configuration was aligned separately, and shape variables were obtained via a Generalized Procrustes Analysis, which translates each configuration to a common origin, scale, and rotation, removing the non-shape variation [81]. Semi-landmarks describing the contours of the bones were declared and aligned using the Procrustes distances minimization criterion [82] between each specimen curve and the consensus curve [83]. Given that the dorsal and ventral views of the skull have bilateral symmetry, the shape of each side was inferred using a bilateral symmetry analysis [84]. The mean shape of both sides was used as a shape variable in the subsequent analysis. Shape variables were obtained as coordinates, and the size estimator called centroid size (CS) as the square root of the sum of distances from each landmark to the centroid [85]. The geometric morphometric analysis was carried out in the package “geomorph” v. 4.0.1 [86], in the R software v.3.6.0 [87].

To evaluate module partitioning in the lateral view of the skull, the correlation between the two modules was tested with a Partial Least Squares (PLS) analysis, which calculates the correlations between the configuration matrices by the first PLS vectors from each matrix. The statistical significance of these correlations was evaluated using permutation tests after 1000 random repetitions [88]. Furthermore, a modularity test was conducted to calculate the coefficient of Covariation Ratio (CR), which indicates the independence between modules considering a null model of total integration. Both analyses were performed using the R package “geomorph” v. 4.0.1 [86].

Analyses were conducted to test for differences in the CS and shape variables between phylogroups, henceforth referred to as “lineages” for morphometric analysis. Sex was included as a factor since our results of external morphology and previous studies [24,72] suggested female-biased sexual size dimorphism in the genus *Corynorhinus*. Thus, a Procrustes ANOVA model was applied to test the effect of CS and sex nested in lineage on the shape variance of each module and view. This was calculated as the Procrustes distance variance with respect to the mean shape of each factor [89]. Given the inequality of sample size between levels of factors and the loss of independence among the shape data [90], the significance of the *F*-statistic for each factor and variable was tested using a resampling test with 1000 replicates of the residuals of the model in the R package “RRPP” v. 0.4.2 [91]. As a *post hoc* test, the differences in shape between lineages separated by sex were explored using paired comparisons between the group means, and their significance was tested by permutation testing and Bonferroni’s correction, comparing the observed Procrustes distance with that obtained from the random assignment of observation to groups in the R package “Morpho” v. 2.7 [92].

Differences between mean shape configurations of lineages were evaluated with Canonical Variate Analysis (CVA) of a previous Principal Components Analysis. To avoid bias caused by sample size differences among lineages, the first five and first ten principal components (PCs) were selected as shape variables in males and females, respectively. This difference in the number of PCs selected between the sexes was because we had fewer males than females. For those views and modules where differences between lineages were observed, the Mahalanobis distances as well as their P-values were obtained by a permutation test of the original data matrix after 1000 replicates. Shape differences between consensus configurations were obtained to examine shape variation graphically among the lineages. Since the changes were small and therefore difficult to appreciate visually, deformation grids were exaggerated by a magnitude of three to make them more perceptible. The analyses and visualization were performed with the R packages “geomorph” v. 4.0.1 [86], “Morpho” v. 2.7 [92], “MASS” [93], and “ggplot2” [94].

The CS variation among lineages was determined using a linear model. Each lineage was separated by sex, and pairwise comparisons were conducted among lineages after 1000 replicates on the residuals of the model in the R package “RRPP” v. 0.4.2 [91].

#### Nomenclatural acts

The electronic edition of this article conforms to the requirements of the amended International Code of Zoological Nomenclature, and hence the new names contained herein are available under that Code from the electronic edition of this article. This published work and the nomenclatural acts it contains have been registered in ZooBank, the online registration system for the ICZN. The ZooBank LSIDs (Life Science Identifiers) can be resolved and the associated information viewed through any standard web browser by appending the LSID to the prefix ""http://zoobank.org/"". The LSID for this publication is: urn:lsid:zoobank.org:pub:E0214CE5-E65B-4246-8D18-5039C2884F97. The electronic edition of this work was published in a journal with an ISSN, and has been archived and is available from the following digital repositories: PubMed Central, LOCKSS.

## Results

### Population genetic analysis

**Fig 2:**
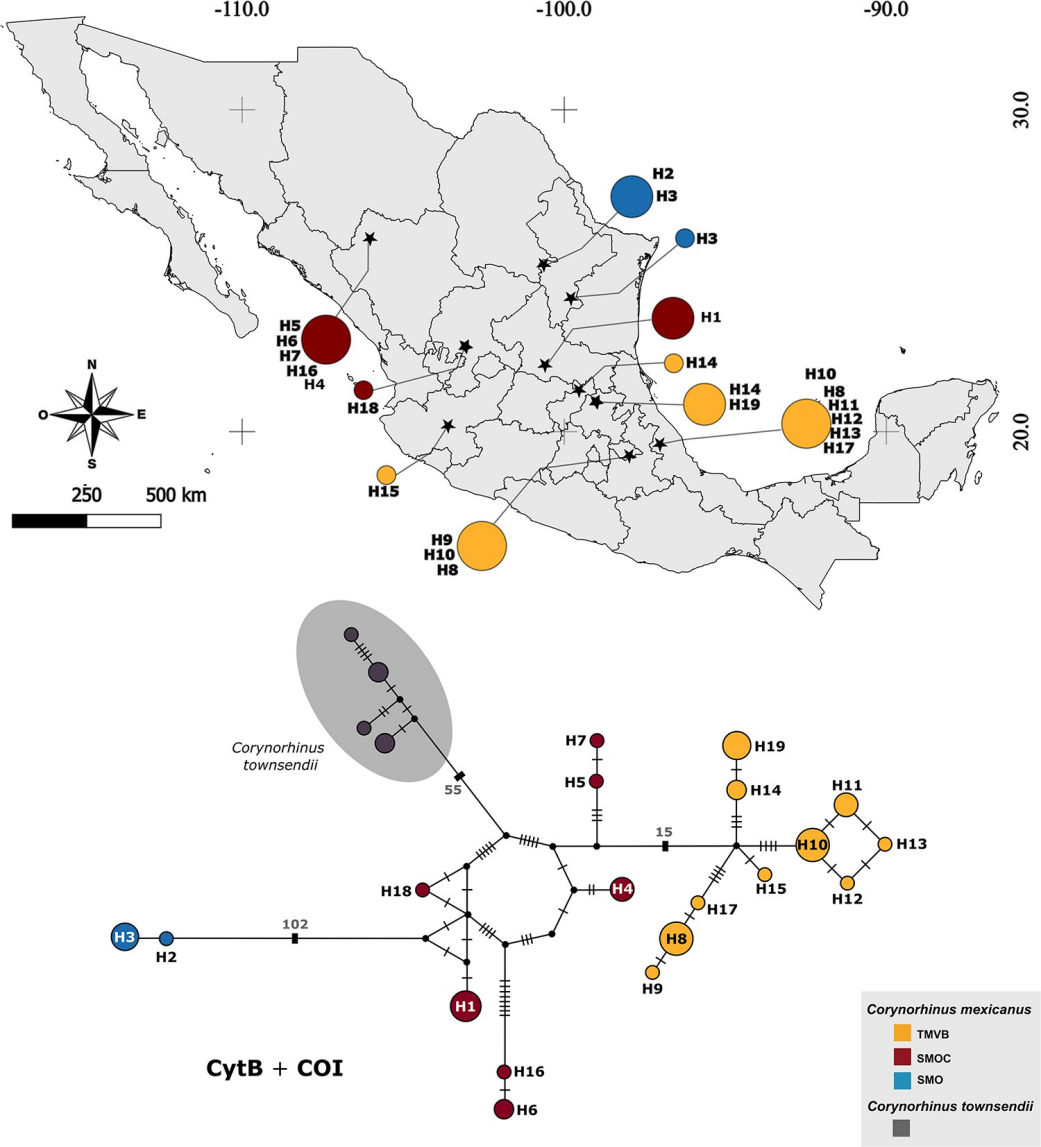
Network of haplotypes of the mitochondrial concatenated Cyt-*b*+COI. The map shows the locations of the DNA samples analyzed, as well as the identity of the haplotypes detected in each location. The SMOC haplogroup is shown in red, the SMO haplogroup in blue, and the TMVB haplogroup in yellow. The size of the circles is proportional to the number of samples present in the haplotype and black lines indicate the observed mutational steps. The Mexican administrative boundaries layer was downloaded from the GADM (https://gadm.org/download_country_v3.html).

From 53 sequences of mtDNA COI, 50 of Cyt-*b*, and 46 of RAG2, we identified 15, 16, and 11 haplotypes, respectively (GenBank# from OQ405113 to OQ405288). For 49 concatenate sequences of the mtDNA COI and Cyt-*b* genes, 23 haplotypes were identified (Fig 2 and Fig B of S2 Appendix).

The Cyt*-b* and COI-Cyt-*b* haplotypes of *C*. *mexicanus* were clustered in three geographical regions, corresponding to i) the northern SMO, ii) SMOC, including haplotypes from San Luis Potosí, and iii) TMVB, including localities from Hidalgo and Queretaro. The haplogroup SMO showed 102 mutational steps with respect to the haplogroups SMOC and TMVB, while 15 mutational steps were found between these two latter groups (Fig 2). In the haplotypes of the concatenated sequences, all samples of *C*. *townsendii* were clustered into a single haplogroup and showed 55 mutational steps with respect to three haplogroups of *C*. *mexicanus*. This number of mutational steps was lower than the number of mutation steps found between haplogroups SMO and SMOC-TMVB (Fig 2). This same pattern of fewer mutation steps was observed in the non-concatenated Cyt-*b* and COI haplotype networks (Fig B of the S2 Appendix). For COI only, two haplogroups were found: SMO and SMOC-TMVB. For RAG2, no geographical pattern was evident. Moreover, the RAG2 haplotypes of *C*. *townsendii* were mixed with those of *C*. *mexicanus* (Fig B of S2 Appendix).

**Fig 3:**
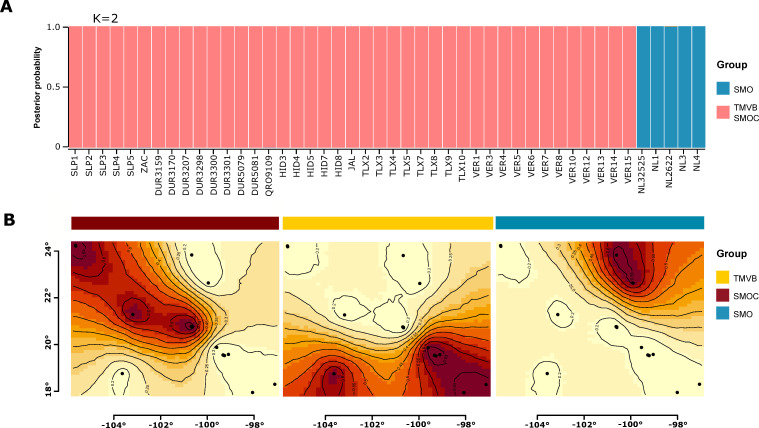
Population genetic structure results. (A) STRUCTURE results when k = 2. (B) GENELAND results with the identity of the haplogroups found in the haplotype network colored with the same color code (see Fig 2).

Genetic structure analyses recovered similar grouping patterns as mitochondrial haplotype data. For concatenated sequences (COI-Cyt-*b*) and by single mitochondrial genes, STRUCTURE detected two genetic groups (K = 2). The first group only included samples from Nuevo Leon state, which corresponded to the haplogroup of SMO obtained in the haplotype networks. The second group included samples from Durango, Zacatecas, San Luis Potosí, Jalisco, Tlaxcala, Queretaro, Hidalgo, and Veracruz, corresponding to the haplogroups from SMOC and TMVB obtained in the haplotype networks (Fig 3). In contrast, GENELAND detected three genetic groups, corresponding to haplogroups found with the Cyt*-b* sequences and COI-Cyt-*b* concatenate sequences in the haplotype networks of these genes (Fig 3).

AMOVA analyses revealed that 84.27% of the total variation was shared among three population groups (SMOC, TMVB, and SMO), but only 9.23% was shared among populations within groups and 6.5% within populations. A similar pattern was observed when considering two groups, given that 85.17% of the total variance was shared between SMO and SMOC-TMVB, but only 11.67% was shared among populations within groups and 3.15% within populations. Both analyses showed a high fixation index, with significant evolutionary divergences both within (Fsc = 0.58 and 0.78) and among (Fct = 0.84 and 0.85) groups (Table 2).

**Table 2:** Summary of analysis of the molecular variance results.

Hypothesis	Source of variation	d. f.	sum of squares	Variance components	% Variation	Fixation index	p-value
Two groups	Among groups	1	461.667	48.68067	85.17	Fct 0.85171	0.023
SMO vs SMOC-TMVB	Among populations within groups	8	245.794	6.67288	11.67	Fsc 0.78728	< 0.001
	Within populations	36	64.908	1.80301	3.15	Fst 0.96845	< 0.001
	Total	45	772.370	57.15656			
Three groups	Among groups	2	624.548	23.37649	84.27	Fct 0.84274	0.003
SMO vs SMOC vs TMVB	Among populations within groups	7	82.913	2.55907	9.23	Fsc 0.58666	< 0.001
	Within populations	36	64.908	1.80301	6.50	Fst 0.93500	< 0.001
	Total	45	772.370	27.73856			

**Fig 4:**
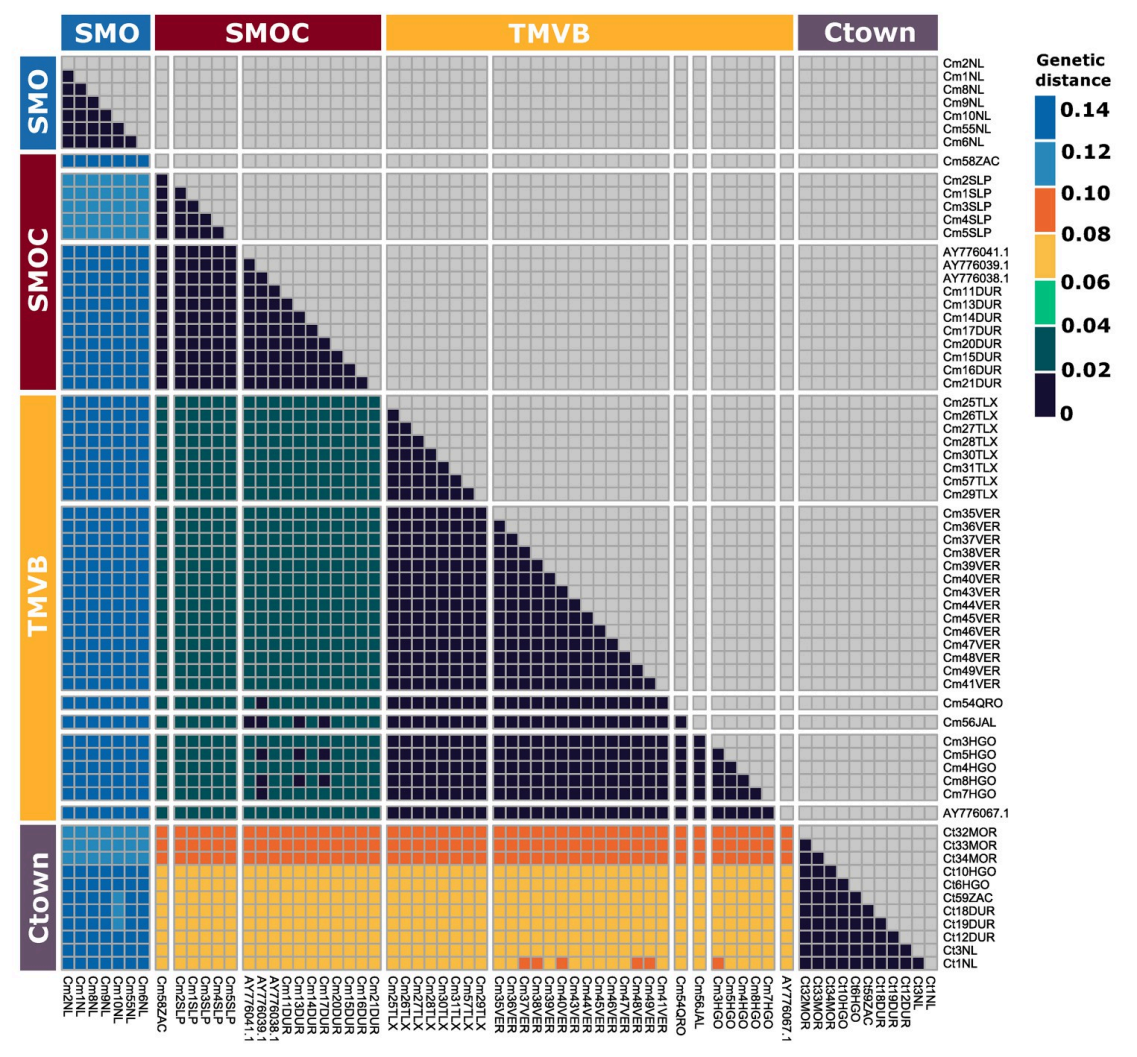
Heatmap of genetic distances. Genetic distances were calculated from 828 bp of the Cyt-*b* gene. Samples were grouped according to haplogroups inferred by GENELAND. In order to compare the magnitude of genetic distance, samples of *C*. *townsendii* were included. The codes of the samples analyzed and their metadata are detailed in Table A of S1 Appendix.

Pairwise genetic distances using Cyt-*b* differed among the SMO, SMOC, and TMVB (Fig 4), with the samples from SMO showing a greater distance relative to those from the TMVB (12.8 ± 0.4%) and SMOC (13.87 ± 0.2%), while the lowest distance was between the TMVB and SMOC (2.7 ± 0.3%). Moreover, the genetic distances between *C*. *townsendii* and all groups of *C*. *mexicanus* (12.7 ± 0.2 SMO; 7.8 ± 0.6% TMVB, and 8.1 ± 0.6% SMOC), were even lower than distances observed between SMO vs. SMOC and SMO vs. TMVB (Fig 4).

#### Phylogenetic analysis, mitochondrial phylogenomics, and species tree

The BioNJ ILD analysis showed topological conflict between nuclear and mitochondrial genes (*p*< 0.001). Both the BioNJ ILD and NJ LILD tests showed that most of the topological incongruence occurred in clades that correspond to *C*. *townsendii*, and the SMOC and TMVB groups of *C*. *mexicanus* (Fig C of S2 Appendix). The Templeton test suggested that RAG2 was causing topological incongruence. A posterior congruence analysis with NJ LILD excluding RAG2, showed topological incongruence (p< 0.05) at the terminal branches only (common ancestors between each individual), while ancient branches had p> 0.05 (Fig D of the S2 Appendix). In addition, the BIONJ ILD test showed no significant topological incongruence in the mitochondrial concatenated tree (p = 0.08). For these reasons, phylogenetic inferences were conducted using mitochondrial data only.

**Fig 5:**
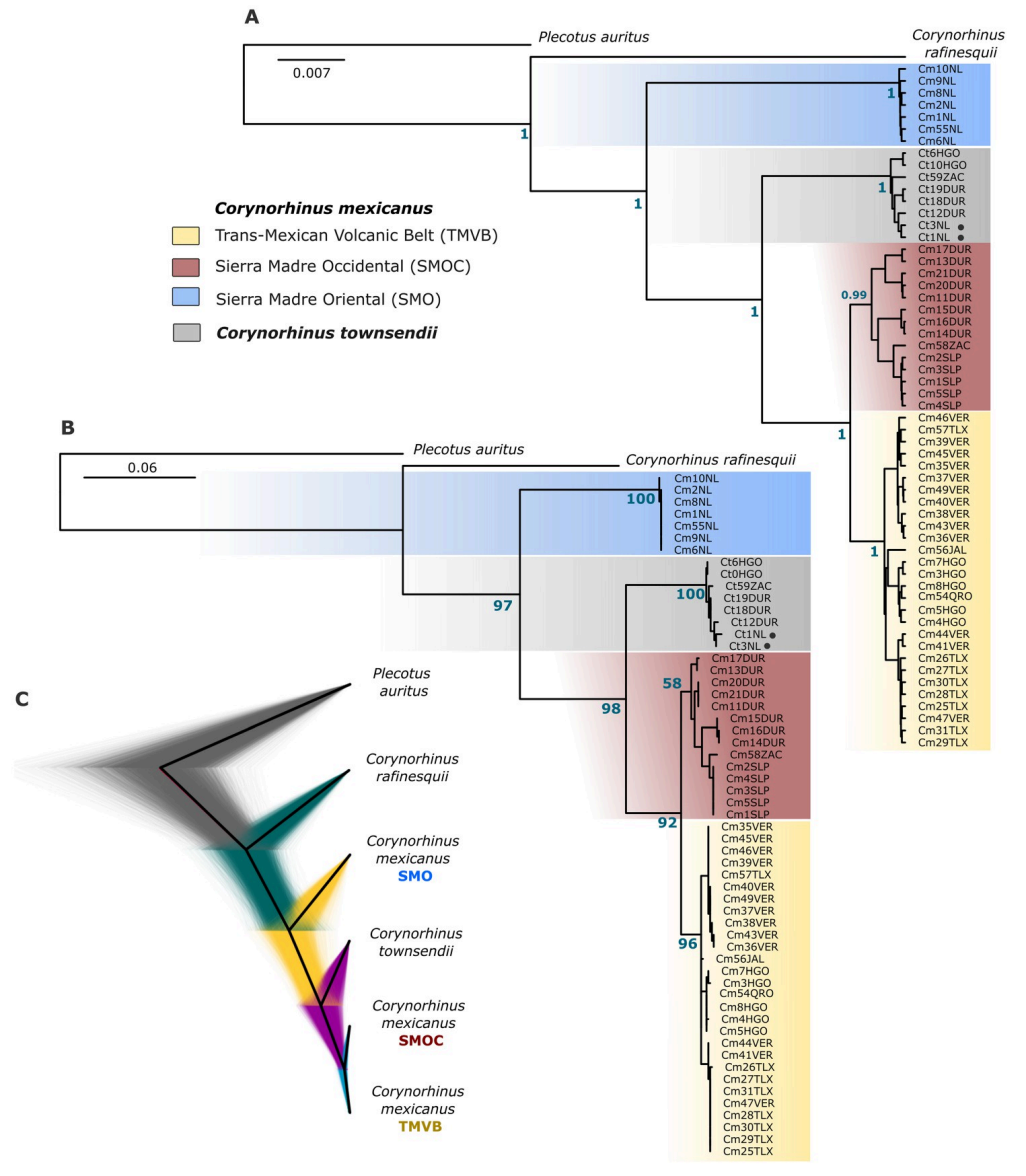
Phylogenetic position of *C*. *mexicanus* within the genus *Corynorhinus*. Trees inferred by Bayesian inference (A) and maximum likelihood (B) using the best partition schemes. The branch support values (posterior probability and ultrafast bootstraps) are shown in blue. Lineages are colored with the same color code used for the mitochondrial haplogroups. Samples of *C*. *townsendii* collected in sympatry with *C*. *mexicanus* are denoted by black dots. For geographical location, see Table A of the S1 Appendix. Panel (C) shows the most common topology of the species tree inferred with *BEAST and displayed in DensiTree.

**Fig 6:**
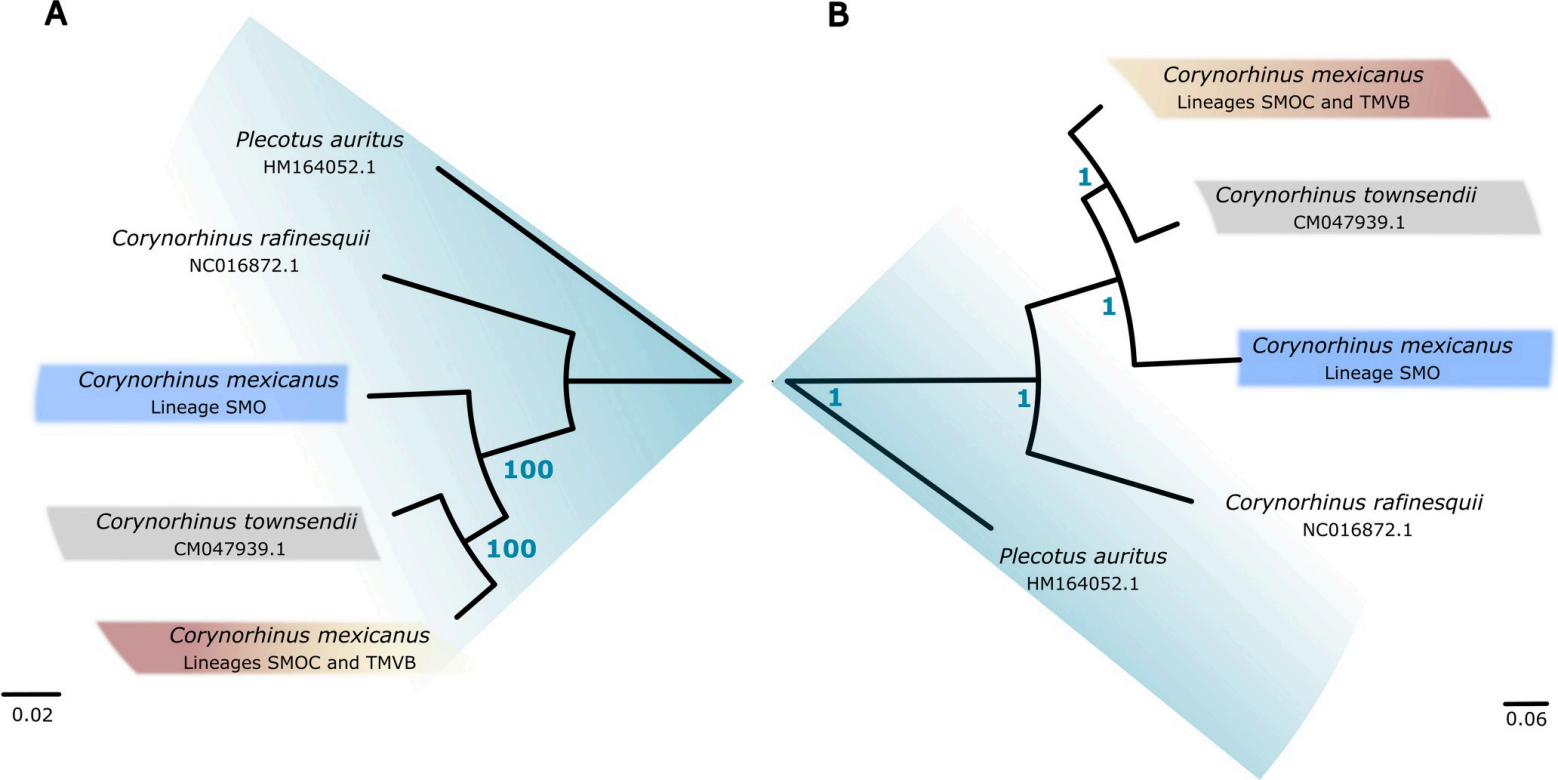
Mitochondrial phylogenomics. Trees inferred by maximum likelihood (A) and Bayesian inference (B) using the mitochondrial genome. The branch support values (ultrafast bootstraps and posterior probability) are shown in blue.

Trees inferred by Maximum Likelihood (ML) and Bayesian Inference (BI) with both partition schemes (*Best scheme* and *Codon position*) showed that *C*. *mexicanus* is a polyphyletic group composed of three phylogroups embedded in two majors, not directly related, monophyletic groups (Fig 5). One major group comprised the phylogroups SMOC and TMVB that share a common ancestor with *C*. *townsendii*. However, in this group, the phylogenetic trees showed a discrepancy in the monophyly of the phylogroups SMOC and TMVB. Some topologies showed that the phylogroups SMOC and TMVB are individually monophyletic lineages, whereas for other topologies, the phylogroup SMOC is within TMVB and both conform a single monophyletic lineage. The other group was composed of samples from the SMO and was recovered as the sister group of the clade *C*. *mexicanus* (SMOC and TMVB phylogroups)–*C*. *townsendii*. This same arrangement was shown in the phylogenetic trees reconstructed using the mitochondrial genome. Both inference methods (Maximum likelihood and Bayesian) indicated, with strong branch support, that *C*. *mexicanus* comprises two major and not directly related clades (Fig 6).

The inferred species tree showed seven different topologies. The first topology grouped 97.83% of the inferred trees (Fig 5), the second grouped about 1.29%, and the remaining five grouped less than 0.83% of trees. The first topology was identical to the phylogenies inferred by Maximum likelihood and Bayesian methods with mitochondrial data (Cyt-*b–*COI). Discrepancies between the first and second topologies were due to the position of the SMO group, which was associated in the first with *C*. *townsendii* + *C*. *mexicanus* (SMOC and TMVB lineages) but was linked to *C*. *townsendii* in the second.

### Divergence time estimates

**Fig 7:**
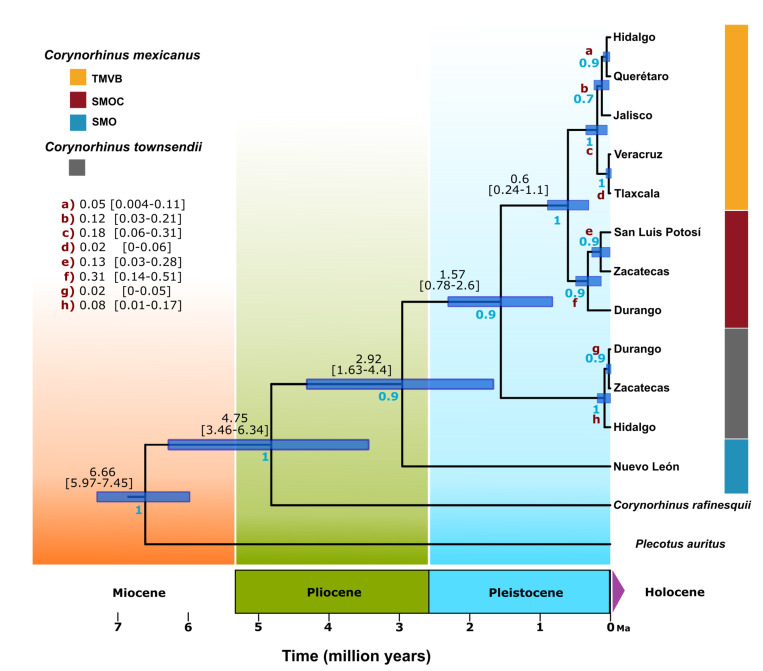
Divergence times estimation within the genus *Corynorhinus* and *Corynorhinus mexicanus* lineages. Ultrametric tree constructed using Cyt-*b* sequences. Each node shows in brackets and blue bars the interval with the highest posterior density (95% HPD) and the mean time that the most recent common ancestor occurred (TMRCA). The posterior probability of each clade is shown in blue. Color of clades corresponds to the same colors used for the mitochondrial haplogroups and phylogenetic lineages.

The divergence time estimated for segregation of the genus *Corynorhinus* from the genus *Plecotus* was around 6.6 million years ago (Ma) (95% highest posterior density [HPD] 5.88–7.36) during the Upper Miocene. However, the origin of the crown group of *Corynorhinus* was estimated at around 4.28 Ma (HPD 3.16–5.49) during the Pliocene, corresponding to a splitting of the clades *C*. *rafinesquii* and *C*. *mexicanus + C*. *townsendii*. The SMO lineage of *C mexicanus* separated from *C*. *mexicanus* (SMOC and TMVB) *+ C*. *townsendii* at around 2.85 Ma (HPD 1.95–3.82) at the transition between the Pliocene and Pleistocene. *Corynorhinus townsendii* separated from *C*. *mexicanus* (SMOC and TMVB) at around 1.55 Ma (HPD 0.98–2.16) during the early Pleistocene. Within *C*. *mexicanus*, the lineages of SMOC and TMVB split at around 0.6 Ma (HPD 0.33–0.9) during the middle Pleistocene (Fig 7).

#### External morphometric analysis

Sexual variation in forearm, tibia, and ear length was found with the t-test (Table A in S4 Appendix). Cohen’s D indicated a moderated sexual variation in forearm length, with females exhibiting larger forearms than males (42.23 ± 1.14 mm vs. 40.96 ± 0.99mm, respectively). For tibia and ear lengths, Cohen’s D indicated a moderate sexual variation, with females having larger values (Table A in S4 Appendix). No sexual variation was observed in tragus length and the number of interfemoral ridges. The interaction lineage*sex did not show statistical differences in the two-way ANOVA of external morphological characters, suggesting that sexual variation is uniform throughout the lineages (forearm, *F*_2_,_145_ = 2.68, *p* = 0.07; tibia, *F*_2,107_ = 2.52, *p* = 0.08; ear, *F*_1,102_ = 0.051, *p* = 0.95).

The comparison among lineages provided by the one-way ANOVA and one-way Kruskal-Wallis showed that only forearm length in males varied among lineages (*F*_2,71_ = 4.733, *p* = 0.01) (Table B-C of S4 Appendix). According to the *post hoc* test, only males of the TMVB showed statistical differences with respect to the SMOC lineage (*q* = 3.78, *p <* 0.05, *D* = 0.72), with the bats of the TMVB (forearm: 41.2 ± 1.03 mm) having larger forearms than those of the SMOC (forearm: 40.5 ± 0.6 mm). The females did not show differences between lineages.

#### Geometric morphometric analysis

Modularity and integration analyses of cranial characters indicated a modular structure. The correlation among modules was low, but significant in all cranial views (dorsal, *r* = 0.44 *p* = 0.001; ventral, *r* = 0.6 *p* = 0.001; lateral, *r* = 0.74 *p* = 0.001). In addition, the Covariation Ratio (CR) coefficient values were < 1 and significant for all cranial views (dorsal, CR = 0.74, *p* = 0.018; ventral, CR = 0.75, *p* = 0.001; lateral, CR = 0.74, *p* = 0.001). These results supported our decision to keep the module partition.

**Fig 8:**
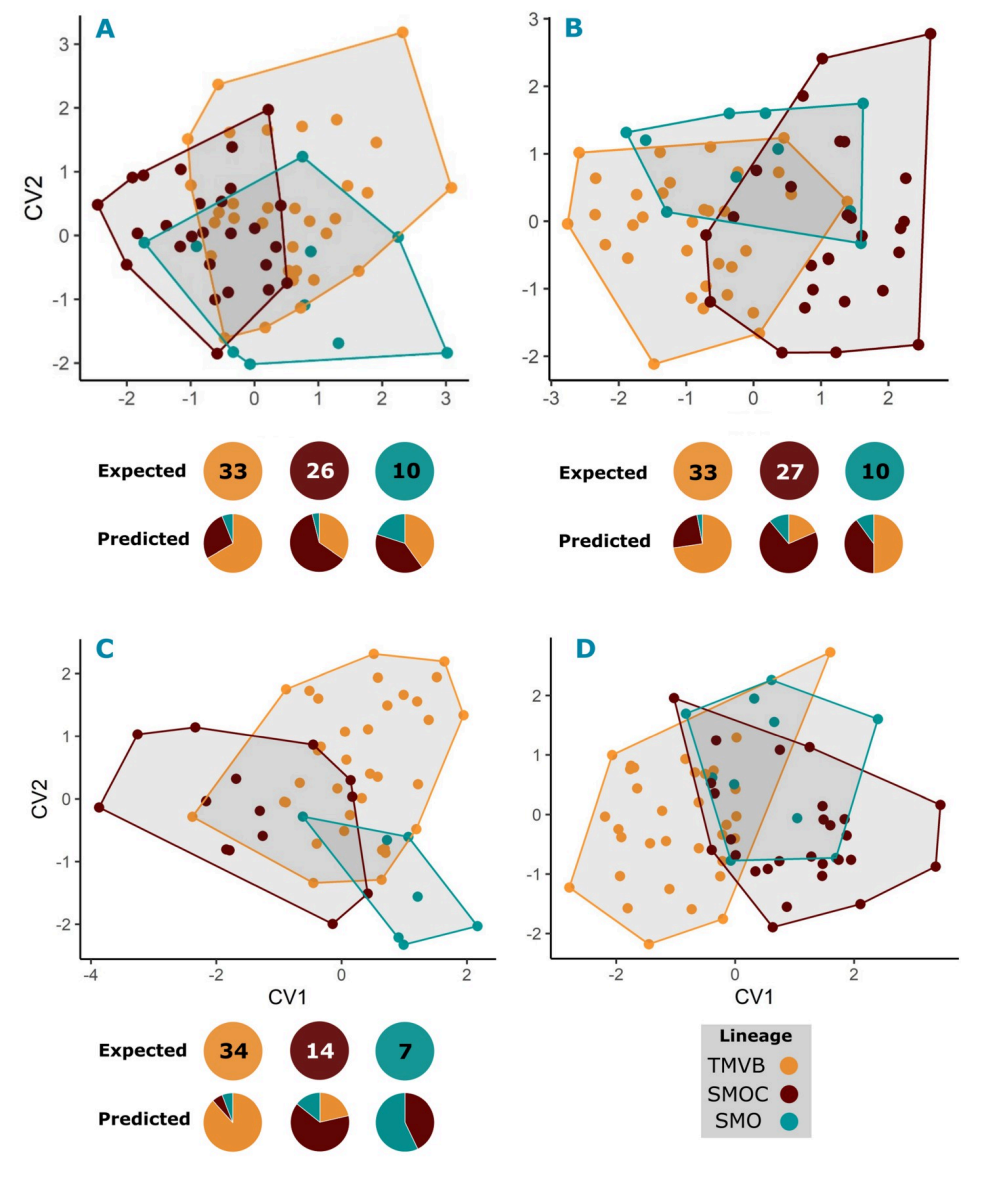
Shape differentiation of cranial modules among lineages. Canonical Variate Analysis (CVA) plots showing the trends of shape differentiation within the lineages: Dorsal view of the rostrum in females (A); lateral view of basicranial module in females (B) and males (C); and lateral view of the rostrum in females (D). For the comparisons with significant differences (A, B and C), the expected values are shown, corresponding to the sample size for each lineage, as well as the predicted values, corresponding to the proportion of individuals assigned to each group. The figures and percentages of the cross-validation are detailed in Table D of the S4 Appendix.

**Fig 9:**
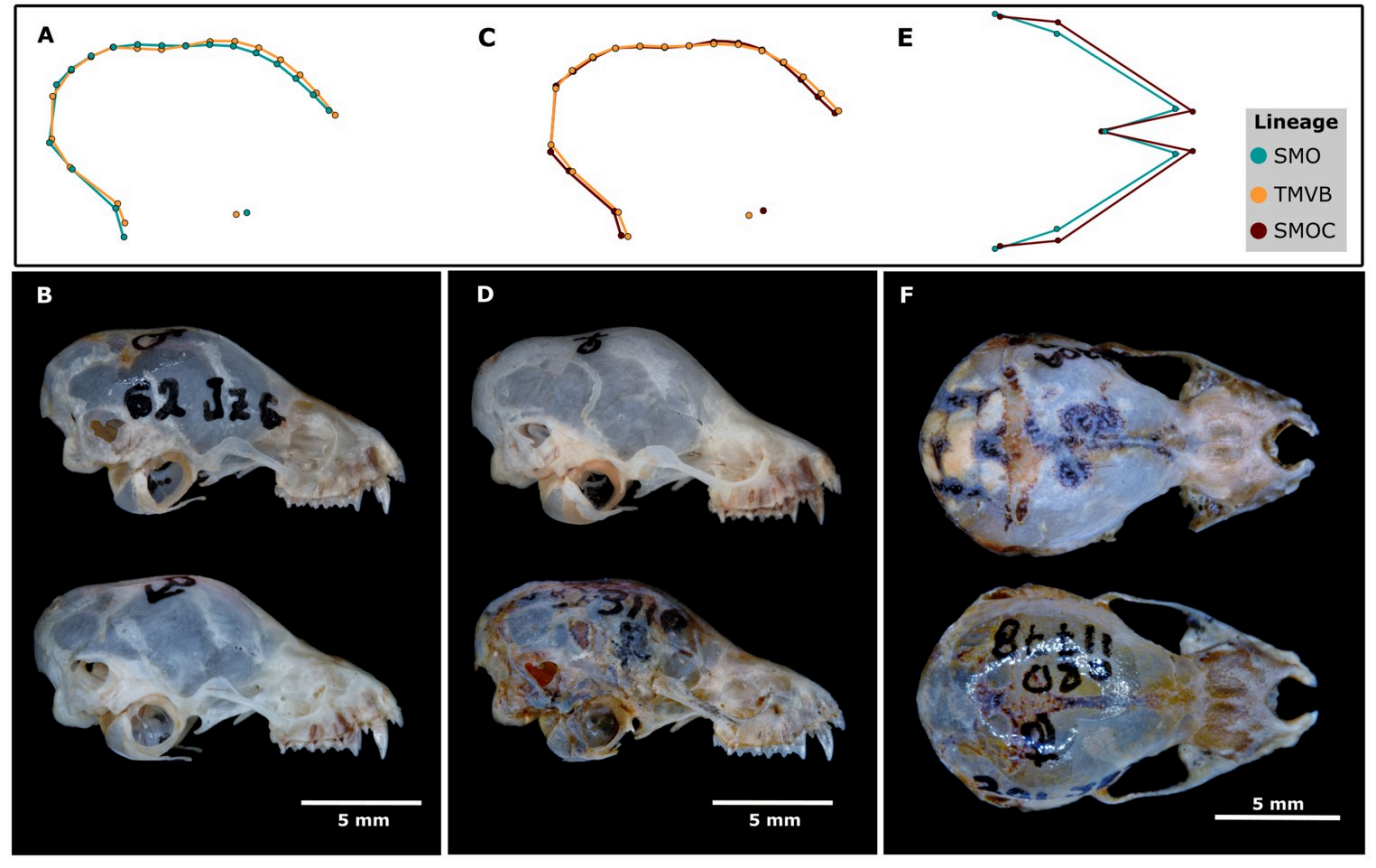
Morphological comparison of the dorsal region of the rostrum and lateral region of the braincase among the lineages. The comparison between consensus shape (upper) and digital photographs (bottom) is shown. Basicranial shape variation is shown for males (A, B) and females (C, D). The male specimens presented (B) correspond in descending order to CRD11777 (SMO) and ENCB3807 (TMVB), whereas the females (D) correspond to CRD0838 (TMVB) and CRD3110 (SMOC). Rostral shape variation in females is also presented (E, F). The female specimens presented (F) correspond in descending order to CRD3304 (SMOC) and CRD11778 (SMO).

**Fig 10:**
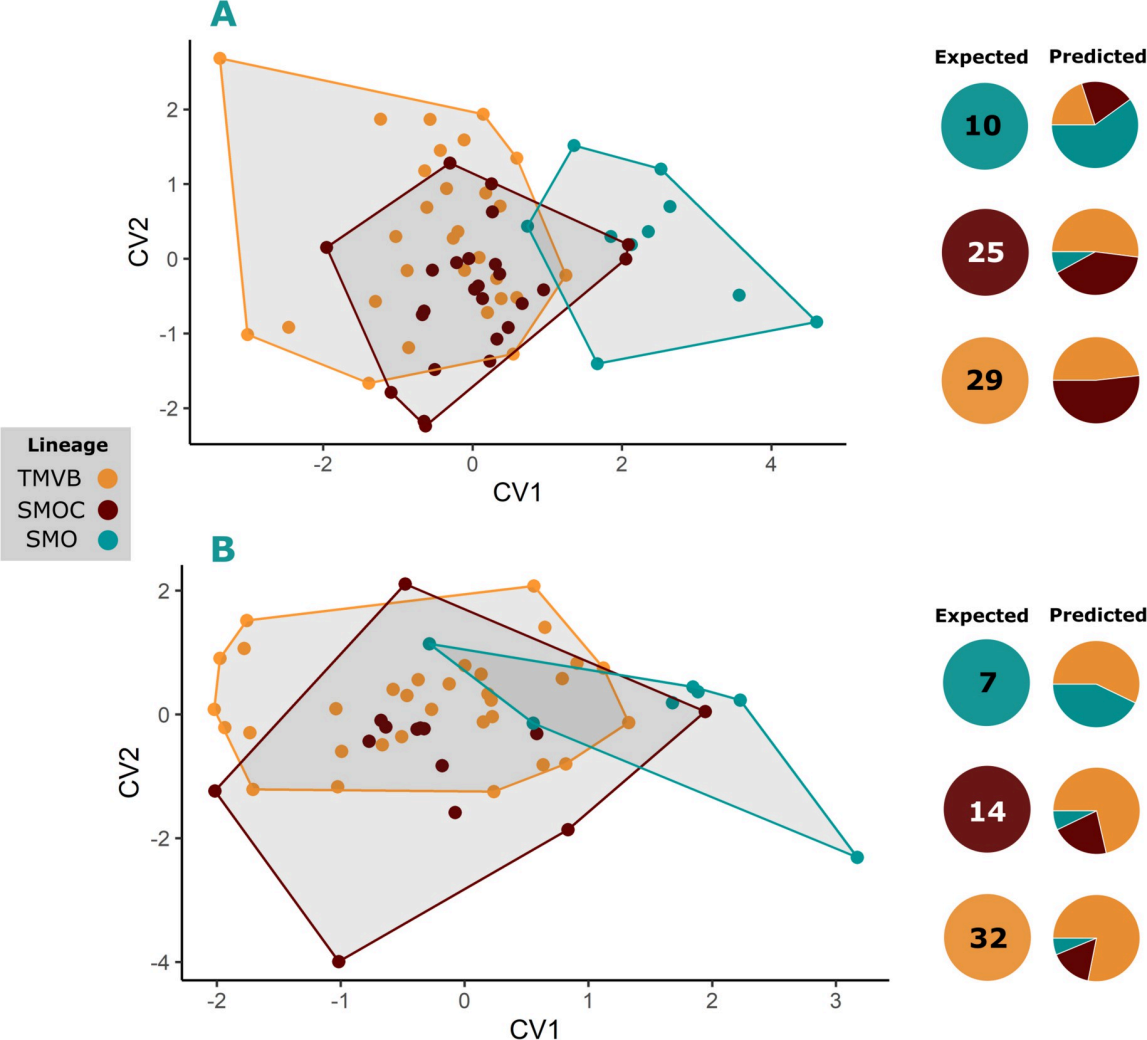
Shape differentiation of mandible within lineages in both sexes. Canonical Variate Analysis (CVA) plots showing the trends of mandible shape differentiation within lineages in females (A) and males (B). For each comparison, the expected values are shown, corresponding to the sample size for each lineage, and the predicted values, corresponding to the proportion of individuals assigned to each group. The figures and percentages of the cross-validation are detailed in Table D of the S4 Appendix.

**Fig 11:**
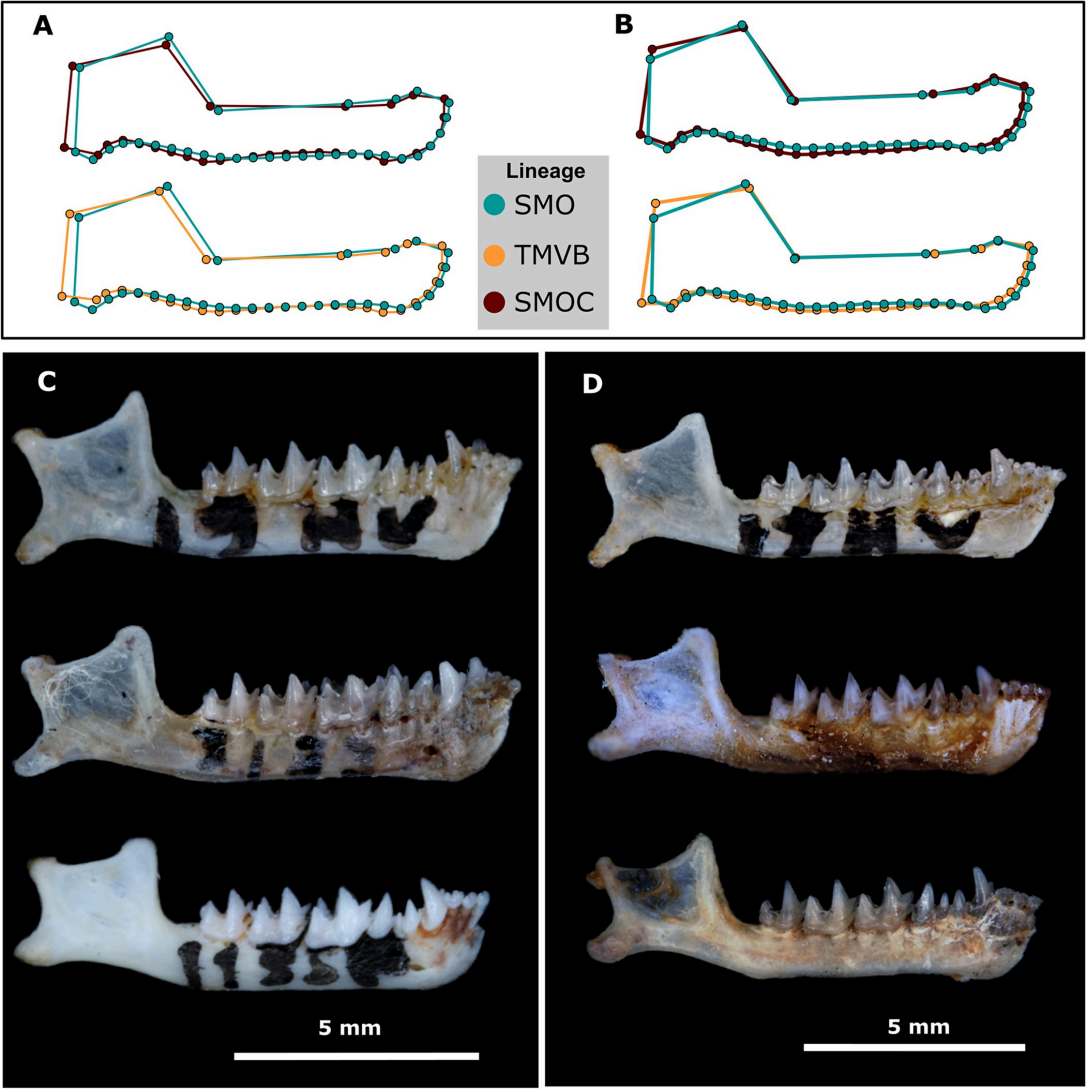
Comparison of mandibular morphologies among lineages in both sexes. The jaws of males (A and C) and females (B and D) are shown. The male specimens presented in C correspond in descending order to: CRD11774 (SMO), CRD3125 (SMOC), and ENCB42211 (TMVB). The female specimens presented in D correspond in descending order to: CRD11773 (SMO), MZFC13051 (TMVB), and CRD3303 (SMOC).

The Procrustes ANOVA revealed that the molecular lineages separated by sexes and centroid size (CS) both have an effect on shape variation. The differences in shape among the lineages were observed in most of the modules, except for the basicranial ventral view, whereas CS had a minimum effect on shape among the lineages (2.7% to 13.4%) (Table 3). Pairwise comparisons revealed that females of the SMOC and SMO presented differences in the dorsal view of the rostrum (*dP* = 0.04, *p* = 0.03) (Fig 8), mainly in terms of the width of the nasal bone and length of the maxillary (Fig 9). In the lateral view of the basicranium, differences were observed in females from the TMVB and SMOC (*dP* = 0.013, *p* = 0.002), as well as in males from the SMO and TMVB (*dP* = 0.013, *p* = 0.02) (Fig 8). These differences were focused mainly on the degree of bulky frontal and parietal bones (Fig 9), but the mandible also showed differences between lineages (Fig 10). Females from the SMO showed greater differences between condylar and coronoid processes than those from the SMOC (*dP* = 0.016, *p* = 0.02) and TMVB (*dP* = 0.015, *p* = 0.04). This same phenomenon was observed in males from the SMO and TMVB (*dP* = 0.017, *p* = 0.02) (Fig 10 and Fig 11). For the rest of the modules and views, although there were no statistical differences, the morphospace showed divergent patterns between some lineages. For example, in females from the SMO and SMOC, the shape coordinates of the lateral view of the rostrum were on opposite sides of the morphospace (Fig 8).

**Table 3:** Shape variation explained by factors “Sex” nested in “Lineage”.

Module	Factor	df	SS	MS	R^2^	F	Z	p-value
Lateral basicranium	CS	1	0.003	0.003	0.027	3.700	3.194	0.001*
	Sex: lineage	5	0.009	0.002	0.093	2.517	4.528	0.001*
	Residuals	119	0.083	0.001	0.880			
	Total	125	0.094					
Lateral	CS	1	0.005	0.005	0.037	4.934	3.258	0.001*
Rostrum	Sex: lineage	5	0.008	0.002	0.059	1.567	1.751	0.045*
	Residuals	119	0.124	0.001	0.903			
	Total	125	0.137					
Dorsal	CS	1	0.001	0.001	0.065	8.666	3.73	0.001*
Basicranium	Sex: lineage	5	0.001	0.0002	0.065	1.731	1.97	0.028*
	Residuals	116	0.015	0.0001	0.870			
	Total	122	0.018					
Dorsal	CS	1	0.041	0.041	0.134	20.28	4.867	0.001*
Rostrum	Sex: lineage	5	0.03	0.006	0.099	3.008	2.888	0.004*
	Residuals	116	0.234	0.002	0.767			
	Total	122	0.305					
Ventral	CS	1	0.006	0.006	0.071	8.944	5.382	0.001*
Basicranium	Sex: lineage	5	0.003	0.001	0.039	0.978	0.022	0.519
	Residuals	113	0.078	0.001	0.891			
	Total	119	0.088					
Ventral	CS	1	0.011	0.011	0.106	14.252	6.343	0.001*
Rostrum	Sex: lineage	5	0.006	0.001	0.055	1.475	1.75	0.04*
	Residuals	113	0.087	0.001	0.839			
	Total	119	0.104					
Mandible	CS	1	0.002	0.002	0.027	3.332	2.91	0.003*
	Sex: lineage	5	0.005	0.001	0.07	1.713	2.192	0.018*
	Residuals	110	0.059	0.001	0.902			
	Total	116	0.065					

Regarding the CS comparison between lineages, in the dorsal view of the braincase, males of the SMO lineage presented lower CS compared to those of the TMVB lineage (d = 0.002, p = 0.01). For the dorsal view of the rostrum, only the females of the TMVB presented higher CS compared to those from the SMOC (d = 0.002, p = 0.01) and SMO (d = 0.001, p = 0.034). On the other hand, the CS of the ventral view of the rostrum was lower in males of the TMVB compared to the SMOC lineage (d = 0.003, p = 0.009). In the case of the mandible, only the SMO males presented a higher CS compared to the SMOC males (d = 0.51, p = 0.029).

Modules, factors, and parameters obtained in the Procrustes ANOVA are shown. CS (centroid size). Significant P- values (< 0.05) are indicated with *.

## Discussion

The hypothesis of the presence of cryptic species within *C*. *mexicanus* was evaluated through phylogenetics, population genetics, and morphometric analyses, representing the first attempt to understand the evolutionary history of *C*. *mexicanus* at an intra- and interspecific level. Moreover, the implementation of new molecular markers, analysis techniques, and different kinds of data enhances our understanding of the evolutionary history of this species and the genus as a whole. In this sense, we highlight that, despite the initial inclusion of RAG2, this nuclear marker was ultimately excluded from most of the results since it was not particularly informative in the context of the proposed hypothesis. RAG genes have been considered very useful for inferring phylogenetic relationships among species [95]; however, their low rate of variation and recombination is inappropriate for making phylogenetic inferences in recently diverged species [95] such as those of the genus *Corynorhinus* [33,71].

On the other hand, the analysis of morphological variation in the three lineages of *Corynorhinus mexicanus* indicated that they have high similarity in most of the morphological and morphometric characters analyzed. Nevertheless, some characters such as mandible shape and forearm length presented a morphological divergence among lineages that allow their discrimination.

### Phylogenetic and population genetic analysis

The tree topologies produced by the phylogenetic analyses support three premises: a) *Corynorhinus rafinesquii* is the species with the oldest divergence time of the crown group of *Corynorhinus*, b) *Corynorhinus townsendii* is the species phylogenetically most related to *Corynorhinus mexicanus* (mainly to the lineages of SMOC and TMVB), and c) the populations from the northern SMO recognized as “*Corynorhinus mexicanus*” constitute a monophyletic sister group that differs from the *C*. *mexicanus* + *C*. *townsendii* clade.

The segregation of *C*. *rafinesquii* in the phylogenetics as the first lineage to diverge from the crown group was previously proposed by Piaggio and Perkins [33] and Lack and Van Den Bussche [71], who also argued that *C*. *townsendii* rather than *C*. *rafinesquii* is the species most closely related to *C*. *mexicanus*. The discovery of the group of *C*. *mexicanus* from the SMO is novel, with respect to previous phylogenetic studies conducted with *C*. *mexicanus* and the genus *Corynorhinus*. This novelty is due to previous studies [33,71] not including a geographic representative sample of *C*. *mexicanus*. In addition, the population genetics, genetic distances, and phylogeny results suggest the presence of an undescribed species, which had gone unnoticed due to the lack of samples collected from this mountain system.

The SMO lineage is supported as a different species from *C*. *mexicanus* by both the phylogenetic analyses and population genetics data. STRUCTURE and GENELAND showed a clearly different genetic structure between samples from the SMO and the SMOC-TMVB lineages. According to Hillis et al. [96], these results may also suggest a lack of connectivity between the SMO and TMVB lineages, even though their distribution over the mountain systems of the Trans-Mexican Volcanic Belt and the Sierra Madre Oriental is apparently continuous. Moreover, the genetic distance observed in the Cyt-*b* gene between the SMO and the TMVB (12.8%) or SMOC (13.6%) lineages indicates a high degree of genetic differentiation. According to Bradley and Baker [41], those populations with a genetic distance higher than 11% on *C*yt-*b* sequences should be recognized as full species. In this context, our results suggest that the SMO lineage exhibits a genetic distance higher than 11% in comparison to the SMOC and TMVB lineages. Consequently, it warrants recognition as a distinct species within the genus *Corynorhinus*.

On the other hand, although GENELAND showed that the populations of the SMOC, including San Luis Potosí, and the TMVB represent two genetic groups, the genetic distances (2.7%) suggested that these groups are not different species. The COI haplotype networks do not show a clear integration and segregation of these lineages, suggesting that they are the same. Moreover, the phylogenetic analysis was not conclusive in terms of whether SMOC and TMVB represent one or two monophyletic groups. With this evidence, we cannot propose taxonomic changes for the SMOC and the TMVB populations.

### Divergence time estimation and probable paleo-environment context

The different divergence periods obtained in this study with Cyt-*b* support those estimated by Piaggio and Perkins [33] and Lack and Van Den Bussche [71], who found that the *Corynorhinus* crown group originated around the Pliocene and early Pleistocene. However, the divergence times calculated by Lack and Van Den Bussche [71] are less conservative and older. For example, Piaggio and Perkins [33] proposed that *C*. *townsendii* and *C*. *mexicanus* (the SMOC and TMVB lineages in this study) diverged approximately 1.8 million years ago (Ma), a finding that is very similar to the results of this study (1.55 Ma, [95% HPD, 0.78–2.6]); however, Lack and Van Den Bussche [71] calculated this divergence at 2.97 Ma (95% HPD, 1.61–4.38), which broadly overlaps with estimates found in this study and that of Piaggio and Perkins [33]. In this sense, we proposed that, despite the variance in estimated dates of divergence between these studies, the geological periods (Pliocene and early Pleistocene) in which the probable divergence episodes occurred are the same, and we therefore discuss some hypotheses regarding the cladogenesis and origin of lineages of *Corynorhinus mexicanus* (including the SMO lineage).

The phylogenetic position and divergence time of *C*. *rafinesquii* and the SMO lineage of *C*. *mexicanus* suggests that the crown group of *Corynorhinus* may have its origin in eastern North America as a consequence of vegetation and environmental changes that occurred during the Pliocene (detailed below). This hypothesis is supported by the fossil record of the extinct species *Corynorhinus alleganiensis*, described from samples discovered in the Cumberland Cave in Maryland, USA [97], the age of which corresponds to the Middle Pleistocene, although the presence of supraorbital sulci in the fossil suggests that this species had a basal affinity to the rest of the species of the genus *Corynorhinus* [24].

The Pliocene, during which the divergence of *C*. *rafinesquii* and the SMO lineage occurred, was characterized by an increase in global temperatures, presenting higher maximums than those experienced at present [98]. The sea level rose as a consequence of the melting ice masses at the polar caps [99], and there was a period of drought that caused the emergence of grasslands and great plains in central USA, where a large part of the Megafauna would graze [100]. It has been proposed that, during this period, the sea level rise would presumably have inundated cave systems in the southeastern USA, and large forests were reduced to discontinuous patches, which served as potential refuges for ancestral *Corynorhinus* [71]. This would lead these populations to disperse in search of new refuges, causing fragmentation in the populations and limiting their connectivity, ultimately leading to speciation events.

The current distribution of the three lineages of *C*. *mexicanus*, including the SMO lineage, may be the consequence of changes in the vegetation and environmental conditions that occurred during the Pleistocene and early Holocene. For example, as with *C*. *rafinesquii* and *C*. *alleganiensis*, the SMO lineage of *C*. *mexicanus* would have had a large distribution over the eastern USA, with a subsequent reduction and fragmentation of its distribution during the Pleistocene as seen in other mammalian taxa with current distributions in the eastern USA and northeastern Mexico [101]. Further studies are required to establish which factors have contributed to the current distribution and how the species distribution of *Corynorhinus* has changed over time.

### External morphometric analysis

Size comparison of the external structures by sex between groups indicated that the three lineages (SMO, SMOC, and TMVB) of *C*. *mexicanus* present a high similarity in size. Previously, Handley [24] reported a similar result when analyzing specimens of *C*. *mexicanus* from localities that correspond to the distribution of the genetic groups found in this study. A probable reason for this similarity in body size is the distribution of the three lineages over coniferous-oak forests that are predominantly cold and humid for a large part of the year. This environmental similarity across their distribution areas implies similar metabolisms that could result in convergence in body size [102].

### Morphological divergences among molecular lineages

In general, cranial modules are very similar in shape among lineages separated by sexes. This agrees with Handley [24] who, using traditional morphometrics, states that the cranial morphology of *C*. *mexicanus* presents an incipient geographical variation. Nevertheless, the slight changes in shape found in the rostrum and lateral view of the braincase could suggest that the lineages are subject to different selection pressures that bring about these morphological changes. For example, the variation in the shape of the braincase and rostrum found in the *C*. *mexicanus* lineages suggests possible differences in echolocation. This premise is supported by the fact that the braincase and rostrum morphologies are correlated with the auditory system and echolocation call emission, respectively [103].

Mandible shape was the main source of variation between the two major monophyletic groups of *C*. *mexicanus* (SMO and SMOC-TMVB) and is also the factor that provides the greatest support for proposing the SMO lineage as a different species in a morphological context. This same pattern of change in mandible shape has previously been observed in the species complexes *Glossophaga soricina* [19] and *Pipistrellus pipistrellus-Pipistrellus pygmaeus* [104]. The mandible and diet are two characteristics of the species that have an intimate form-function relationship [105–107]. This association is based mainly on the correlation between the distances and proportions of the coronoid and condylar processes of the mandible and the size of the temporalis and masseter muscles, which account for part of the bite force [106,107]. Generally, species with a bite force that permits them to consume hard prey have more pronounced coronoids located at a greater distance from the condylar process, in addition to thick dental bones [108]. In contrast, species that consume soft prey tend to have a coronoid close to the condylar process and positioned at a similar height, as well as thinner dentaries [106,107].

Unlike the linages SMOC and TMVB, the mandible in males and females of the SMO lineage showed higher placed coronoids with respect to the condylar process, as well as a greater distance between them (Fig 11). These characteristics could suggest that the diet of this lineage may comprise relatively tougher insects than those consumed by the SMOC and TMVB lineages. The diet in *C*. *mexicanus sensu lato* has not been studied in detail; however, it is known that, as with the rest of the species of *Corynorhinus*, the Mexican long-eared bat consumes soft insects, mainly lepidopterans [39,40]. The diversity of Lepidoptera tends to vary along altitudinal, latitudinal, and seasonal gradients [109], so there is likely to be variation among the diets of the rest of the lineages. In addition, knowledge regarding the nocturnal Lepidoptera of Mexico is scarce [109], which makes it even more difficult to determine the possible diet of the SMO and TMVB-SMOC lineages and to assess whether the morphological variation of the mandible is a product of differentiation in the diet. Future dietary studies comparing prey diversity among lineages could shed light on the cause of these morphological changes in the mandible of *C*. *mexicanus*, including in the SMO lineage.

The results of the analysis of variation in shape and size suggest that, despite the morphological divergence in cranial and mandible structures, the differentiation between lineages is barely perceptible, which complicates their discrimination in the field. Although this low magnitude of morphological variation among lineages does not contribute greatly to taxonomic diagnosis, it does suggest possible ecological, physiological, and functional influences that have affected the evolutionary history of the lineages.

### Taxonomic proposal

Family: Vespertilionidae Gray, 1821

Genus: *Corynorhinus* H. Allen, 1865

### *Corynorhinus leonpaniaguae* sp. nov.

urn:lsid:zoobank.org:act:98B459DC-ADF6-4F7A-88A7-8F5FEAC58C10

Vernacular name: León Paniagua’s Big-eared Bat, Murciélago-mula de León-Paniagua **(**Spanish**)**

#### Synonyms

*Corynorhinus macrotis pallescens* Miller, 1897, (Part)

*Corynorhinus megalotis mexicanus* Allen, 1916

*Corynorhinus rafinesquii mexicanus* Miller, 1924, (Part)

*Plecotus rafinesquii mexicanus* Dalquest, 1953, (Part)

*Plecotus mexicanus* Handley, 1959

*Corynorhinus mexicanus* G. M. Allen, 1916, (Part)

#### Holotype

**Fig 12:**
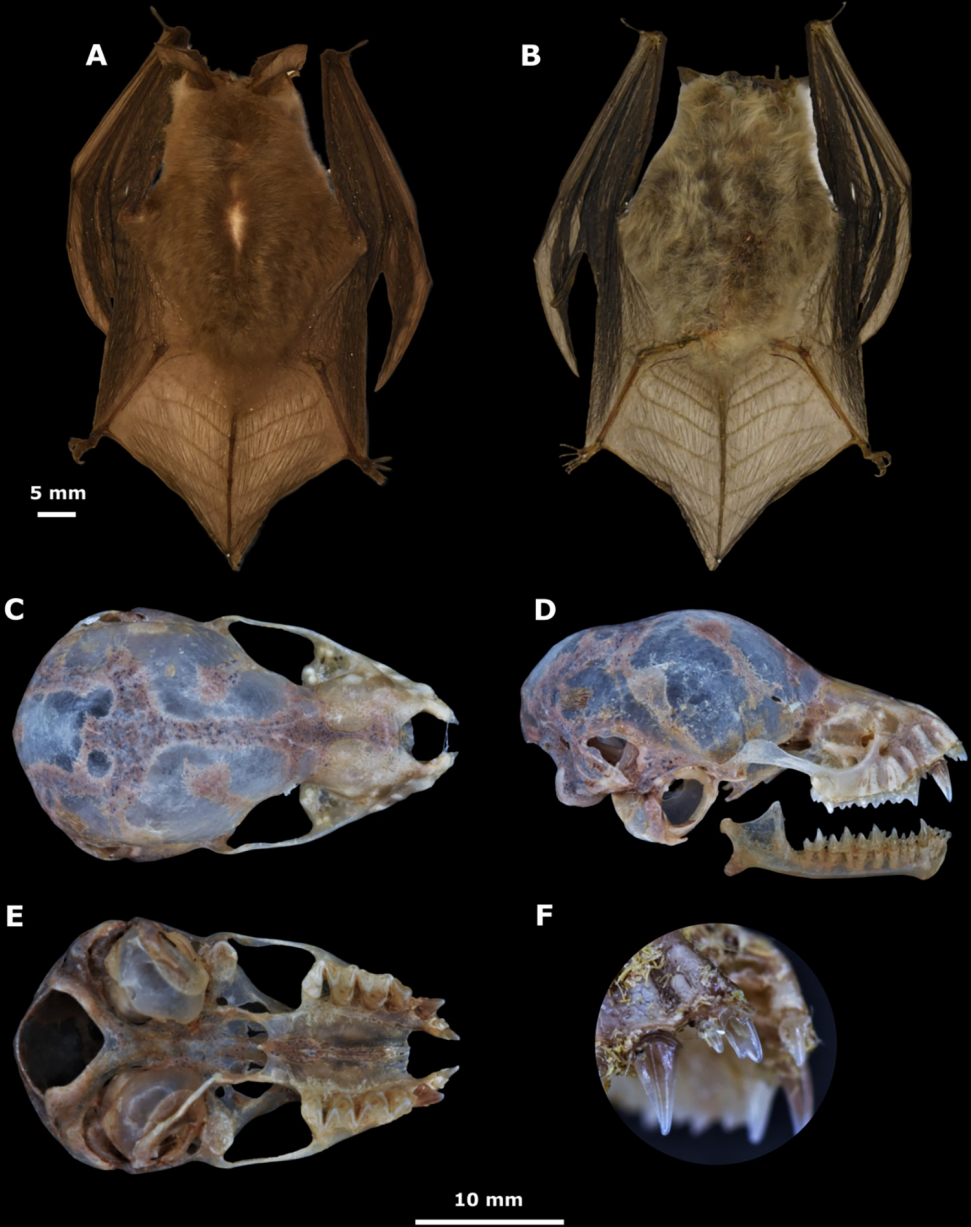
Holotype pictures. Coloration of the dorsal and ventral fur of the holotype (MZFC-M16326) of *Corynorhinus leonpaniaguae* sp. nov. (A, B). Cranium, mandible (C, D, E) and lateral view of the first upper double-cuspid incisor (F) of the holotype.

Colección de Mamíferos, Museo de Zoología Alfonso L. Herrera (MZFC-M), No. MZFC-M16326 is an adult female with skin and skull dry preserved and collected on April 18^th^, 2022, by Juan Cruzado, Silvino Hernández and Issachar L. López-Cuamatzi (Fig 12).

#### Type locality

Cave El Hundido (25.16096, -100.622985; 2072 msnm), 2.5 Km NE from Puerto Grande, Galeana, Nuevo León, Mexico.

#### Etymology

*Corynorhinus leonpaniaguae* is named in honor of Dr. Livia S. León Paniagua, in recognition of her outstanding contribution to the knowledge of the systematics and natural history of Mexican mammals. In addition to being a pioneering woman in Mexican mammalogy, Livia has been a great mentor, dedicated to the training of new scientists. This species name is a noun in the genitive case formed by adding -ae to the stem of the name [110].

#### Habitat and distribution

**Fig 13:**
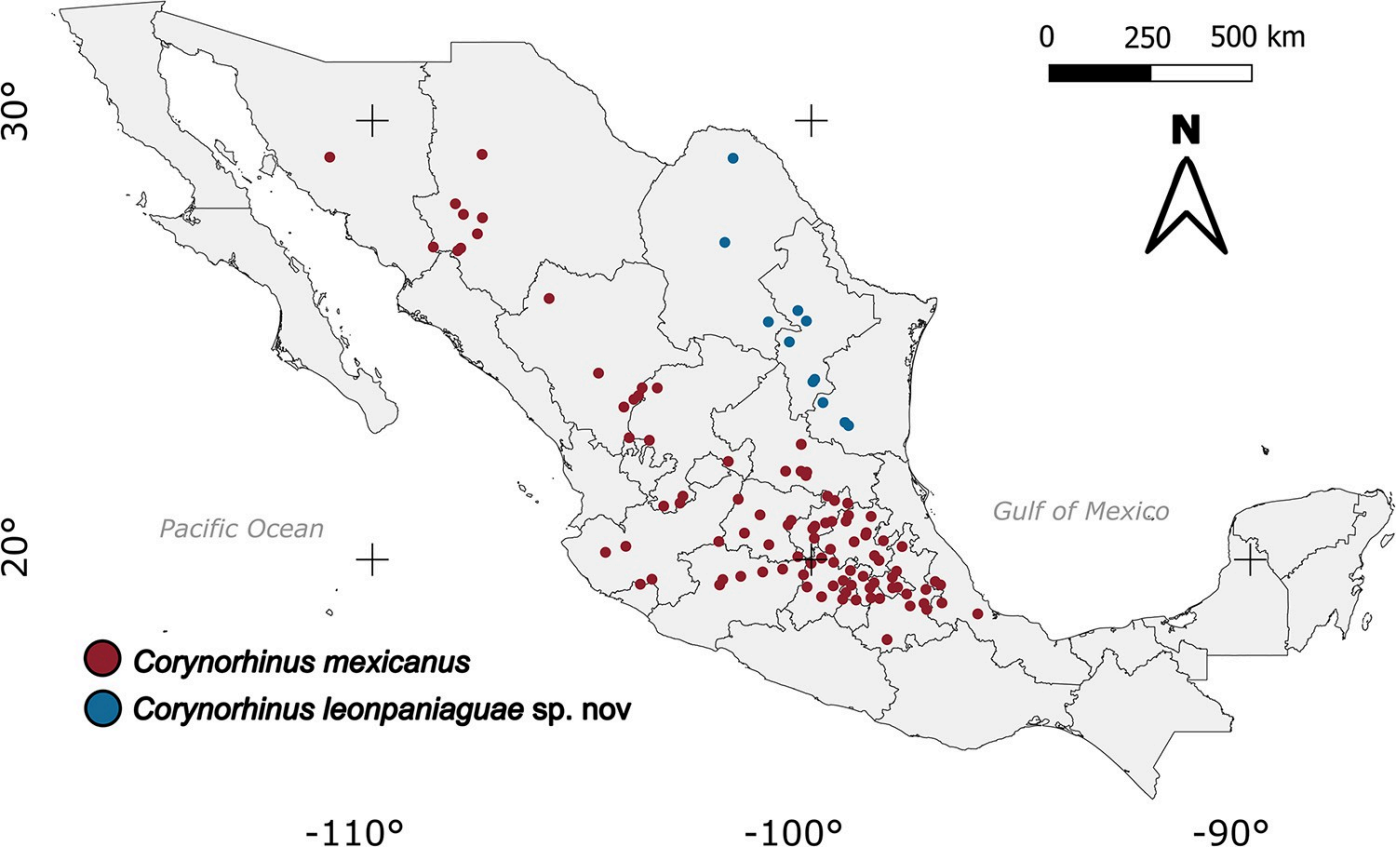
Historical records of the presence of *Corynorhinus mexicanus* and *Corynorhinus leonpaniaguae* sp. nov. The Mexican administrative boundaries layer was downloaded from the GADM (https://gadm.org/downloadcountryv3.html).

*Corynorhinus leonpaniaguae* lives in open pine forests located on slopes and canyons of the northern Sierra Madre Oriental and mountains of the state of Coahuila. Records of the presence of this species occur mainly between 300 and 2000 meters above sea level (masl) in the states of Coahuila, Nuevo León, and Tamaulipas. It is an endemic species of Mexico with distribution restricted to the northeast of the country (Fig 13). In the localities of Galeana, Nuevo León, and Sierra de Zapalinamé in Arteaga, Coahuila, specimens were captured in sites close to forests where globose and cylindrical cacti are present. Other plant species present are *Pinus cembroides*, *Pinus* sp, *Juniperus* sp, *Yucca filifera*, *Y*. *linearifolia*, and *Yucca carnerosana*. The characteristics of the roosts used by this species are unknown; however, in Coahuila, one specimen was captured during the day inside an abandoned mine, and, in Nuevo León, a colony with pregnant females was found in a limestone, dolomite, and gypsum cave.

#### Description and comparison

**Fig 14:**
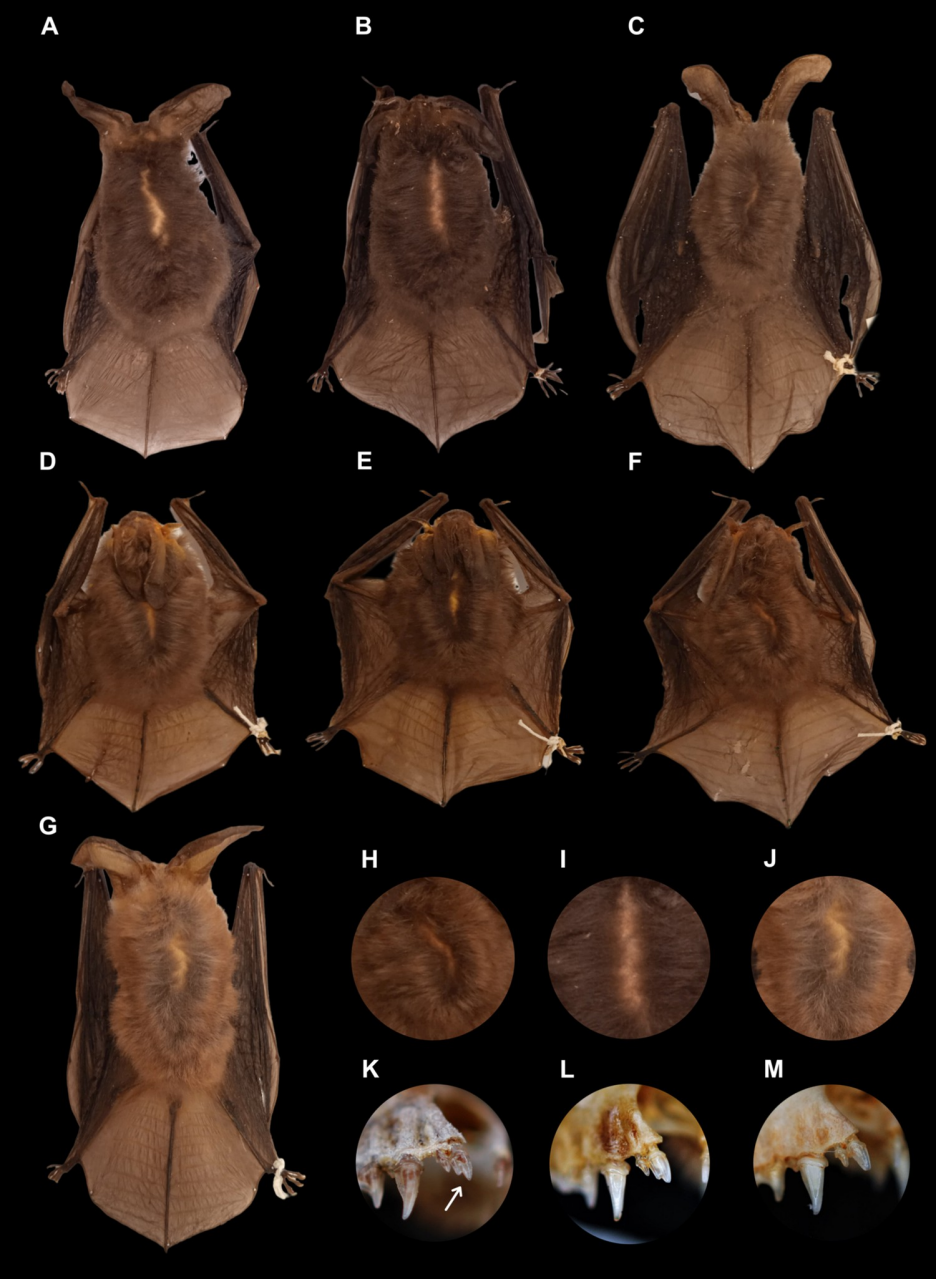
Comparison between *C*. *mexicanus*, *C*. *leonpaniaguae*, and *C*. *townsendii*. Specimens shown correspond to *C*. *mexicanus* from (A) Puebla (ENCB27986), (B) Tlaxcala (ENCB4405), and (C) Durango (CRD11777); C. *leonpaniaguae* from the cave of San Josecito, in Gral. Zaragoza, Nuevo Leon, Mexico (D- CRD11774; E- CRD11778; and F- CRD11777). Specimen of *C*. *townsendii* from Durango (G- CRD4831). Some differences in color bands on the dorsal fur are shown for *C*. *leonpaniaguae* (H), *C*. *mexicanus* (I), and *C*. *townsendii* (J). Double-cuspid on upper incisor tooth (white arrow) observed in *C*. *leonpaniaguae* (K), *C*. *mexicanus* (L), and *C*. *townsendii* (M).

The specimens from Nuevo León present a brownish to grayish colored dorsal fur with dark bases and slightly lighter tips that appear to contrast between bands. However, this contrast is not equivalent to that observed in *Corynorhinus townsendii* (Fig 14). Like *C*. *mexicanus* and *C*. *rafinesquii*, *C*. *leonpaniaguae* presents a double cuspid in the first upper incisor (Figs 12 and 14). Its dental formula is i 2/3, c 1/1, p 2/3, and m 3/3, with a total of 36 teeth in its adult stage. The average values of cranial measurements and external measurements for females and males are reported in Table 4.

**Table 4:** Morphological measurements of external structures obtained in *Corynorhinus leonpaniaguae*.

	Females				Males			
	Mean	SD	Range	CV	Mean	SD	Range	CV
Maximum length	14.87	0.34	14.19–15.20	2.28	15.10	0.31	14.8–15.68	2.05
Zygomatic width	7.94	0.36	7.05–8.37	4.60	8.08	0.24	7.79–8.38	3.01
Postorbital width	3.36	0.08	3.24–3.49	2.43	3.36	0.12	3.17–3.54	3.64
Width of the braincase	7.73	0.27	7.33–8.07	3.43	7.72	0.34	7.35–8.38	4.44
Maxillary toothrow length	4.74	0.08	4.67–4.94	1.78	4.69	0.07	4.60–4.80	1.47
Width between upper molars	5.82	0.11	5.66–5.96	1.88	5.74	0.16	5.57–5.97	2.78
Total length	91.59	5.73	84.37–102.79	6.25	90.56	6.00	82.08–101.14	6.62
Tail length	43.37	6.15	34.95–55.75	14.19	40.12	3.53	35.20–44.91	8.79
Ear length	28.66	1.24	27.37–30.43	4.33	28.43	1.39	27.13–31.30	4.89
Tragus length	10.89	0.93	9.54–12	8.54	10.23	0.80	8.69–10.79	7.80
Forearm length	41.91	1.48	38.71–43.94	3.54	40.52	0.99	39.54–42.08	2.44

Mean, standard deviation (SD), range, and coefficient of variation (CV) values are shown. Measurements are reported in millimeters (mm).

In external appearance, it is similar to and is almost indistinguishable from *C*. *mexicanus* specimens from the Sierra Madre Occidental (SMOC). *Corynorhinus leonpaniaguae* is distinguished from *C*. *townsendii* because the latter presents a tragus of > 13 mm and a naked uropatagium with ten or more interfemoral grooves, while the former presents a tragus of < 13 mm and a hairy uropatagium with nine or fewer interfemoral grooves. Moreover, *C*. *townsendii* usually has a larger forearm (39 to 47 mm) and cranium maximum length (15.2 to 17.3 mm) than *C*. *leonpaniaguae*. In external appearance, *C*. *leonpaniaguae* is also similar to *C*. *rafinesquii*; however, *C*. *leonpaniaguae* has bicolored ventral fur with brown bases and light tips, while *C*. *rafinesquii* has more contrasting fur due to the presence of hairs with black bases and white tips.

#### Geographic variation

With the restricted distribution and limited sample size, it was not possible to detect morphological variation associated with geography. Only two haplotypes of Cyt-*b* sequences have been detected within *Corynorhinus leonpaniaguae*.

#### Subspecies

*Corynorhinus leonpaniaguae* is a monotypic species.

#### Natural history

There is no published information documenting the natural history of this species. Some observations made during the fieldwork of this study suggest certain aspects of the biology of *Corynorhinus leonpaniaguae*. In two expeditions conducted in August 2021 and April 2022 at the type locality of this species, several specimens of *Myotis thysanodes*, *Corynorhinus townsendii*, *Leptonycteris nivalis*, *Idionycteris phyllotis*, and *Antrozous pallidus* were captured. These specimens were captured at the entrance of the El Hundido Cave both entering and exiting the cave. This suggests that *Corynorhinus leonpaniaguae* may share its roosts with these species. Of those mentioned above, *M*. *thysanodes* and *C*. *townsendii* were the only species, apart from *Corynorhinus leonpaniaguae*, that were captured on both field trips, indicating that all three species are probably residents of the cave.

In August 2021, four juvenile male individuals were captured, indicating that weaning of these specimens had occurred in June-July, which is similar to that reported for *C*. *mexicanus* [39]. In April 2022, twelve adult females in an advanced state of pregnancy were captured, suggesting that births probably occur between the end of April and mid-May. All specimens captured in May and August presented a considerable number of ectoparasitic flies, presumably of the *Trichobius corynorhini* species.

**Fig 15:**
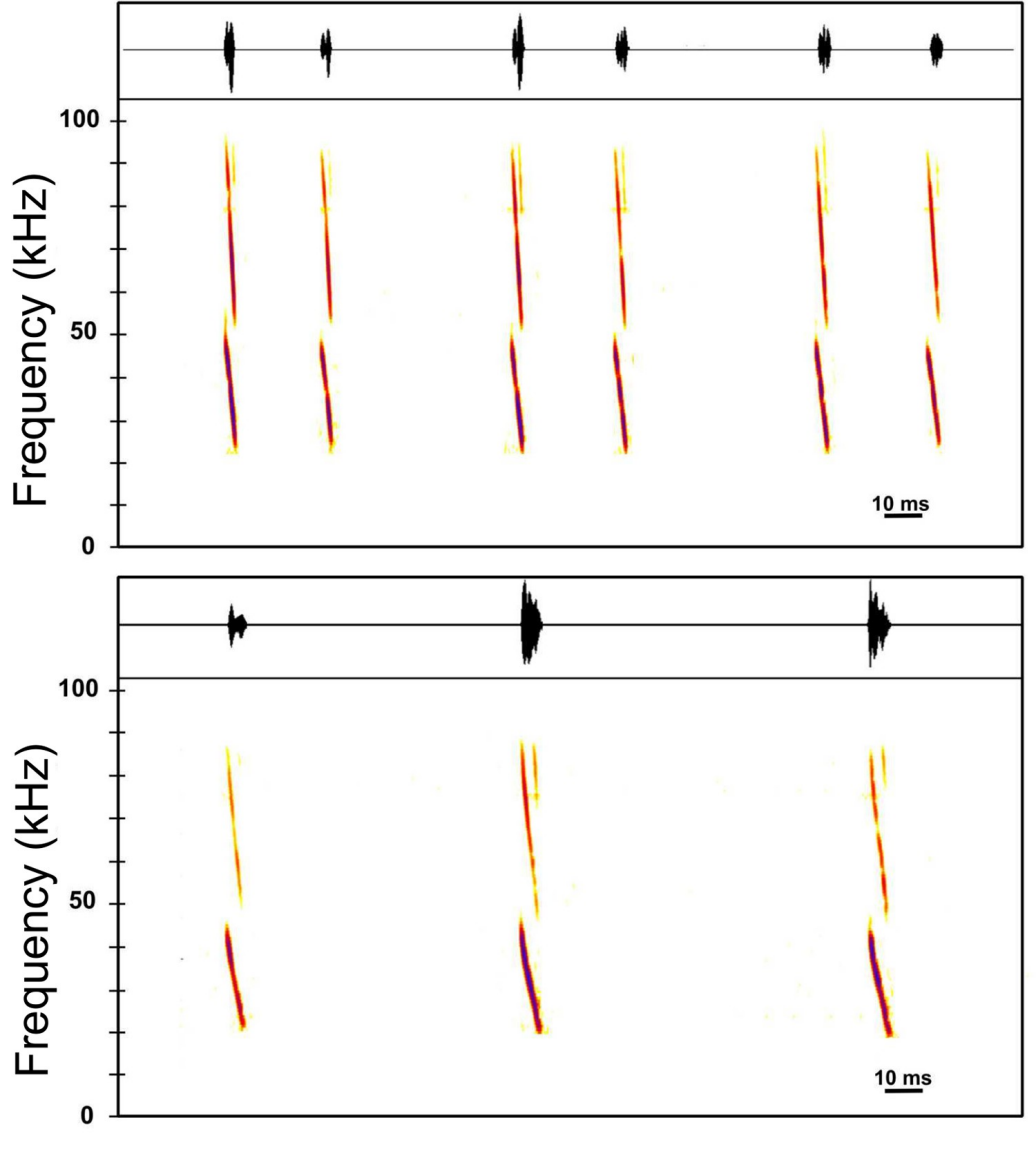
Sonotypes depicting echolocation pulses of *Corynorhinus leonpaniaguae*. Sonotype I (upper panel) comprising combinations of two pulses, and sonotype II (lower panel) comprising single pulses of longer duration.

The acoustic characteristics of *Corynorhinus leonpaniaguae* were obtained from ten specimens recorded using the hand-release technique and an Echo Meter Touch 2 Pro bat detector. Recordings were analyzed with the Batsound® software using Hanning window and 2048 of FFT size. Two sonotypes composed of modulated and harmonic pulses were observed from the recordings (Fig 15). The first sonotype consisted of combinations of two pulses repeated serially with a longer time interval between combinations than between the pulses in the combination. Although two-pulse combinations were predominant, three-pulse combinations were observed in some specimens. The second sonotype was characterized by the absence of pulse combinations and the presence of modulated pulses of longer duration. Details of frequency, duration, and interval are presented in Table 5. Differences in acoustic characteristics among the species of *Corynorhinus* are still unknown.

**Table 5:** Spectro-temporal characteristics of the echolocation pulses of *Corynorhinus leonpaniaguae*.

Sonotype		Fmin (kHz)	Fmax (kHz)	FHA (kHz)	Dur (ms)	IP (ms)	IPC (ms)
I	mean	21.30	48.20	36.40	3.00	22.10	52.90
n = 100	SD	1.15	1.32	0.81	0.37	1.27	5.98
	CV	5.40	2.70	2.20	12.30	5.80	11.30
II	mean	21.70	45.70	35.40	4.70	73.80	-
n = 23	SD	0.73	1.44	1.04	0.73	23.68	-
	CV	3.40	3.20	2.90	15.60	32.10	-

Mean, standard deviation (SD), and coefficient of variation (CV) values of two sonotypes are shown. Abbreviations: Sample size (n), minimum frequency (Fmin), maximum frequency (Fmax), frequency of highest amplitude (FHA), duration (Dur), interval between pulses (IP), and interval between pulse combinations (IPC).

## Conclusion

Based on phylogenetic relationships, Cyt-*b* genetic differences, genetic population structure and shape differences in mandible, we propose that *C*. *mexicanus* is a cryptic species composed of two taxonomic entities. This proposal is supported by the operative criterion to delimit species according to the genetic and phylogenetic species concept and by accumulative and congruence criteria established by integrative taxonomy. Therefore, we propose that *C*. *mexicanus sensu stricto* corresponds to the lineages of SMOC and TMVB, whereas the SMO lineage corresponds to an undescribed and unnamed species. Accordingly, we describe and name a new *Corynorhinus* species, *C*. *leonpaniaguae*.

Taxonomy and systematics are key for the biological conservation and protection of poorly studied and negatively perceived groups. This is one of the reasons why, for bats, taxonomic and systematics studies are more required today. Thanks to scientific and technological progress, “The Age of Discovery is not over for chiropteran taxonomists” [3], and it is through these studies and interdisciplinary collaboration that it is possible to recognize, classify, and protect bat biodiversity. In this sense, we recommend *C*. *mexicanus* and *C*. *leonpaniaguae* should be managed separately and the identification of threats and evaluation of population health of Mexican bats species should be carried out taking into consideration this taxonomic proposal. On the other hand, for *C*. *mexicanus* we recommend more research in order to elucidate if both lineages (SMOC and TMVB) should be managed separately.

## Supporting information

S1 Appendix GenBank accession numbers and information about the specimens reviewed. (DOCX)

S2 Appendix Complementary information regarding DNA extraction, PCR protocols and phylogenetic analysis. (DOCX)

S3 Appendix Complementary information regarding DNA extraction, assembly and annotation of the mitochondrial genome, and phylogenomic analysis. (DOCX)

S4 Appendix Complementary information regarding geometric morphometric analysis. (DOCX)
