# Supplementary material for: Molecular and morphological data suggest a new species of big-eared bat (Vespertilionidae: Corynorhinus) endemic to northeastern Mexico
Source: PLoS One. 2024 Feb 21;19(2):e0296275. doi: 10.1371/journal.pone.0296275 (PMC10881012; doi:10.1371/journal.pone.0296275)
Supplement: S1 Appendix — (DOCX) [file pone.0296275.s001.docx]

**Molecular and morphological data suggest a new species of big-eared bat (Vespertilionidae: *Corynorhinus*) endemic to northeastern Mexico**

**S1 Appendix**

**Scientific Collections Acronyms**

**CRD** Colección de Mamíferos, Centro Interdisciplinario de Investigación para el Desarrollo Integral Regional, Unidad Durango, Instituto Politécnico Nacional

**MZFC-M** Colección de Mamíferos, Museo de Zoología “Alfonso L. Herrera”, Facultad de Ciencias, Universidad Nacional Autónoma de México.

**CNMA** Colección Nacional de Mamíferos, Instituto de Biología, Universidad Nacional Autónoma de México.

**ENCB**  Colección Mastozoológica, Escuela Nacional de Ciencias Biológicas, Instituto Politécnico Nacional.

**TK**  Genetic Resources Collection, Texas Tech University

**UAZ** Colección de Vertebrados, Facultad de Biología, Universidad Autónoma de Zacatecas.

**SLPZ** Colección de Vertebrados, Facultad de Ciencias, Universidad Autónoma de San Luis Potosí.

**IIB** Colección de Mamíferos, Instituto de Investigaciones Biológicas, Universidad Veracruzana.

**UANL** Colección de Mamíferos, Universidad Autónoma de Nuevo León.

**UAEH-CIB** Colección de Mamíferos, Centro de Investigaciones Biológicas, Universidad Autónoma del Estado de Hidalgo.

**UAM-I** Colección de Mamíferos de la Sierra Volcánica Transversal de México, Universidad Autónoma Metropolitana, Unidad Iztapalapa.

**Table A.**  Summary of DNA samples used in genetics analysis. Prefix in Code column indicates the species (Cm- *C. mexicanus*; Ct- *C. townsendii*). In the “**No. of the catalogue”** column, tissues samples obtained in individuals captured in the field are indicated with hyphens (-). Tissue samples donated by scientific collection are indicated by their catalogue number. For the acronyms of scientific collections, see the beginning of this supplementary material.

| **Code** | **#Genbank**  **Cyt-*b*** | **#Genbank**  **COI** | **#Genbank**  **RAG2** | **No. of the catalogue** | **Locality** | **Coordinates** | |
| --- | --- | --- | --- | --- | --- | --- | --- |
|  |  |  |  |  |  | **Latitude** | **Longitude** |
| Cm1COA | - | OQ405115 | - | *-* | Cerca de Huachichil | 25.2061 | -100.8400 |
| Cm11DUR | OQ405184 | OQ405128 | OQ405240 | CRD-3300 | Mina 24, Guanaceví | 25.5732 | -105.5961 |
| Cm13DUR | OQ405185 | OQ405129 | OQ405242 | CRD-5080 | Mina 6, Guanaceví | 25.5390 | -105.5861 |
| Cm14DUR | OQ405186 | OQ405130 | OQ405243 | CRD-3297 | Mina 6, Guanaceví | 25.5390 | -105.5861 |
| Cm15DUR | OQ405187 | OQ405131 | OQ405244 | CRD-3189 | Mina 25, Guanaceví | 25.5732 | -105.5961 |
| Cm16DUR | OQ405188 | OQ405132 | OQ405245 | CRD-3298 | Mina 6, Guanaceví | 25.5390 | -105.5861 |
| Cm17DUR | OQ405189 | OQ405133 | OQ405246 | CRD-5079 | Mina 6, Guanaceví | 25.5390 | -105.5861 |
| Cm20DUR | OQ405191 | OQ405134 | OQ405249 | CRD-3170 | Mina 6, Guanaceví | 25.5390 | -105.5861 |
| Cm21DUR | OQ405192 | OQ405135 | OQ405250 | CRD-3301 | Mina 24, Guanaceví | 25.5732 | -105.5961 |
| Ct12DUR | OQ405226 | OQ405169 | OQ405241 | CRD-5081 | Mina 6, Guanaceví | 25.5732 | -105.5961 |
| Ct18DUR | OQ405227 | OQ405170 | OQ405247 | CRD-8112 | Mezquital | 23.3201 | -104.2745 |
| Ct19DUR | OQ405228 | OQ405171 | OQ405248 | CRD-8111 | Mezquital | 23.3201 | -104.2745 |
| Cm1HGO | - | OQ405159 | OQ405286 | UAEH-CIB2170 | Villa Juárez, PN Los Mármoles | 20.8522 | -99.2228 |
| Cm2HGO | - | OQ405160 | OQ405283 | UAEH-CIB2367 | Jaguey Colorado, PN Los Mármoles | 20.9015 | -99.0516 |
| Cm3HGO | OQ405205 | OQ405161 | OQ405284 | UAEH-CIB2171 | Villa Juárez, PN Los Mármoles | 20.8522 | -99.2228 |
| Cm4HGO | OQ405215 | OQ405162 | OQ405285 | UAEH-CIB2175 | Jaguey Colorado, PN Los Mármoles | 20.8767 | -99.2828 |
| Cm5HGO | OQ405221 | OQ405163 | OQ405282 | UAEH-CIB2359 | Jaguey Colorado, PN Los Mármoles | 20.9015 | -99.0516 |
| Cm7HGO | OQ405222 | OQ405164 | OQ405281 | UAEH-CIB2360 | Jaguey Colorado, PN Los Mármoles | 20.8737 | -99.2741 |
| Cm8HGO | OQ405223 | OQ405165 | OQ405287 | UAEH-CIB2361 | Jaguey Colorado, PN Los Mármoles | 20.8737 | -99.2741 |
| Ct10HGO | OQ405225 | OQ405167 | - | UAEH-CIB2368 | Jaguey Colorado, PN Los Mármoles | 20.9015 | -99.0516 |
| Ct6HGO | OQ405234 | OQ405166 | OQ405288 | UAEH-CIB2252 | Cueva del Basurero, Pacula | 21.0162 | -99.3004 |
| Cm56JAL | OQ405218 | OQ405157 | - | CRD-12062 | El ermitaño, Techaluta | 20.0812 | -103.5965 |
| Ct32MOR | OQ405230 | - | OQ405258 | *-* | El Túnel, Palmira | 18.9613 | -99.2926 |
| Ct33MOR | OQ405231 | - | OQ405259 | *-* | El Túnel, Palmira | 18.9613 | -99.2926 |
| Ct34MOR | OQ405232 | - | OQ405260 | *-* | El Túnel, Palmira | 18.9613 | -99.2926 |
| Cm10NL | OQ405183 | OQ405121 | OQ405239 | *-* | Puerto Grande, Galeana | 25.1610 | -100.6230 |
| Cm1NL | OQ405182 | OQ405113 | - | MZFC-M-16325 | Puerto Grande, Galeana | 25.1610 | -100.6230 |
| Cm2NL | OQ405190 | OQ405114 | - | MZFC-M-16326 | Puerto Grande, Galeana | 25.1610 | -100.6230 |
| Cm55NL | OQ405217 | OQ405120 | OQ405280 | TK-32525 | Cueva San Josecito, Gral. Zaragoza | 23.9618 | -99.9127 |
| Cm6NL | OQ405179 | OQ405116 | OQ405235 | UANL-2622 | Cueva El Infierno, La Camotera | 25.1170 | -100.2143 |
| Cm7NL | - | OQ405117 | OQ405236 | *-* | Puerto Grande, Galeana | 25.1610 | -100.6230 |
| Cm8NL | OQ405180 | OQ405118 | OQ405237 | *-* | Puerto Grande, Galeana | 25.1610 | -100.6230 |
| Cm9NL | OQ405181 | OQ405119 | OQ405238 | *-* | Puerto Grande, Galeana | 25.1610 | -100.6230 |
| Ct1NL | OQ405224 | OQ405173 | - | *-* | Puerto Grande, Galeana | 25.1610 | -100.6230 |
| Ct3NL | OQ405229 | OQ405172 | - | *-* | Puerto Grande, Galeana | 25.1610 | -100.6230 |
| Cm54QRO | OQ405216 | OQ405158 | OQ405279 | TK-9109 | Pinal de Amoles | 21.2015 | -99.5551 |
| Cm1SLP | OQ405174 | OQ405123 | - | SLPZ0006 | Sierra de Álvarez | 22.0582 | -100.6114 |
| Cm2SLP | OQ405175 | OQ405124 | - | SLPZ0043 | Sierra de Álvarez | 22.0771 | -100.6211 |
| Cm3SLP | OQ405176 | OQ405125 | - | SLPZ0050 | Sierra de Álvarez | 22.0813 | -100.6151 |
| Cm4SLP | OQ405177 | OQ405126 | - | SLPZ0031 | Sierra de Álvarez | 22.0964 | -100.6445 |
| Cm5SLP | OQ405178 | OQ405127 | - | SLPZ0032 | Sierra de Álvarez | 22.0964 | -100.6445 |
| Cm25TLX | OQ405193 | OQ405137 | OQ405251 | *-* | Cueva El Bañito, La Malinche | 19.2708 | -98.0194 |
| Cm26TLX | OQ405194 | OQ405138 | OQ405252 | *-* | Cueva El Bañito, La Malinche | 19.2708 | -98.0194 |
| Cm27TLX | OQ405195 | OQ405139 | OQ405253 | *-* | Cueva El Bañito, La Malinche | 19.2708 | -98.0194 |
| Cm28TLX | OQ405196 | OQ405140 | OQ405254 | *-* | Cueva El Bañito, La Malinche | 19.2708 | -98.0194 |
| Cm29TLX | OQ405197 | OQ405141 | OQ405255 | *-* | Cueva El Bañito, La Malinche | 19.2708 | -98.0194 |
| Cm30TLX | OQ405198 | OQ405142 | OQ405256 | *-* | Cueva El Bañito, La Malinche | 19.2708 | -98.0194 |
| Cm31TLX | OQ405199 | OQ405143 | OQ405257 | *-* | Cueva El Bañito, La Malinche | 19.2708 | -98.0194 |
| Cm57TLX | OQ405219 | OQ405136 | OQ405276 | *-* | Cueva El Bañito, La Malinche | 19.2708 | -98.0194 |
| Cm35VER | OQ405200 | OQ405144 | OQ405261 | *-* | Volcancillo, Las Vigas | 19.6232 | -97.0665 |
| Cm36VER | OQ405201 | OQ405145 | OQ405262 | *-* | Volcancillo, Las Vigas | 19.6232 | -97.0665 |
| Cm37VER | OQ405202 | OQ405146 | OQ405263 | *-* | Volcancillo, Las Vigas | 19.6232 | -97.0665 |
| Cm38VER | OQ405203 | OQ405147 | OQ405264 | *-* | Volcancillo, Las Vigas | 19.6232 | -97.0665 |
| Cm39VER | OQ405204 | OQ405148 | OQ405265 | *-* | Volcancillo, Las Vigas | 19.6232 | -97.0665 |
| Cm40VER | OQ405206 | OQ405149 | OQ405266 | *-* | Volcancillo, Las Vigas | 19.6232 | -97.0665 |
| Cm41VER | OQ405207 | OQ405150 | OQ405267 | *-* | Volcancillo, Las Vigas | 19.6232 | -97.0665 |
| Cm42VER | - | - | OQ405268 | *-* | Volcancillo, Las Vigas | 19.6232 | -97.0665 |
| Cm43VER | OQ405208 | OQ405152 | OQ405269 | *-* | Volcancillo, Las Vigas | 19.6232 | -97.0665 |
| Cm44VER | OQ405209 | OQ405151 | OQ405270 | *-* | Volcancillo, Las Vigas | 19.6232 | -97.0665 |
| Cm45VER | OQ405210 | OQ405153 | OQ405271 | *-* | Volcancillo, Las Vigas | 19.6232 | -97.0665 |
| Cm46VER | OQ405211 | OQ405154 | OQ405272 | *-* | Volcancillo, Las Vigas | 19.6232 | -97.0665 |
| Cm47VER | OQ405212 | OQ405155 | OQ405273 | *-* | Volcancillo, Las Vigas | 19.6232 | -97.0665 |
| Cm48VER | OQ405213 | - | OQ405274 | *-* | Volcancillo, Las Vigas | 19.6232 | -97.0665 |
| Cm49VER | OQ405214 | OQ405156 | OQ405275 | *-* | Volcancillo, Las Vigas | 19.6232 | -97.0665 |
| Cm58ZAC | OQ405220 | OQ405122 | OQ405277 | UAZ1 | Susticacán | 22.6107 | -103.1444 |
| Ct59ZAC | OQ405233 | OQ405168 | OQ405278 | UAZ4 | Susticacán | 22.6107 | -103.1444 |

**Table B.**  Summary of metadata of *Corynorhinus mexicanus* museum specimens reviewed for morphological analysis. For the acronyms of scientific collections, see the beginning of this supplementary material. Lineage and sex of specimens are shown. Abbreviations: F, female; M, male; SMOC, Sierra Madre Occidental; TMVB, Trans-Mexcian Volcanic Belt; SMO, Sierra Madre Oriental.

| **No. of catalogue** | **Sex** | **State** | **Lineage** | **Locality** | **Latitude** | **Longitude** | **Collection date** |
| --- | --- | --- | --- | --- | --- | --- | --- |
| CRD736 | F | Durango | SMOC | 9.7 km S, 15.6 km E Vicente Guerrero | 23.64 | -104 | 05/08/1989 |
| CRD737 | F | Durango | SMOC | 9.7 km S, 15.6 km E Vicente Guerrero | 23.64 | -104 | 05/08/1989 |
| CRD838 | F | México | TMVB | 9 km N, 7.5 km E Acambay | 20.03 | -99.8 | 01/04/1986 |
| CRD1155 | M | Durango | SMOC | 4 km N, 7.75 km E La Joya | 23.88 | -104 | 24/05/1995 |
| CRD3082 | F | Durango | SMOC | 5 km NW Guanaceví | 25.95 | -106 | 31/01/2001 |
| CRD3083 | F | Durango | SMOC | 5 km NW Guanaceví | 25.95 | -106 | 31/01/2001 |
| CRD3084 | F | Durango | SMOC | 4 km SW Guanaceví | 25.9 | -106 | 02/02/2001 |
| CRD3085 | F | Durango | SMOC | 4 km SW Guanaceví | 25.9 | -106 | 02/02/2001 |
| CRD3101 | M | Durango | SMOC | Mina "La Mexicana" 3.5 km SSW Guanaceví (Mina 6) | 25.9 | -106 | 13/05/2001 |
| CRD3104 | F | Durango | SMOC | Mina "La Mexicana" 3.5 km SSW Guanaceví (Mina 6) | 25.9 | -106 | 13/05/2001 |
| CRD3106 | M | Durango | SMOC | 3.5 km N, 3 km W Guanaceví (Mina 16) | 25.96 | -106 | 13/05/2001 |
| CRD3110 | F | Durango | SMOC | Mina "La Mexicana" 3.5 km SSW Guanaceví (Mina 6) | 25.9 | -106 | 11/05/2001 |
| CRD3112 | F | Durango | SMOC | 3.2 km NW Guanaceví | 25.95 | -106 | 18/05/2001 |
| CRD3115 | M | Durango | SMOC | 0.9 km N, 1.8 km W Guanaceví (Mina 9) | 25.94 | -106 | 12/05/2001 |
| CRD3125 | M | Durango | SMOC | 2 km WSW Guanaceví (Mina 4) | 25.92 | -106 | 11/05/2001 |
| CRD3126 | F | Durango | SMOC | 2 km WSW Guanaceví (Mina 4) | 25.92 | -106 | 11/05/2001 |
| CRD3148 | F | Durango | SMOC | 1.3 km S, 0.3 km E San Pedro | 25.94 | -106 | 17/05/2001 |
| CRD3170 | F | Durango | SMOC | Mina "La Mexicana" 3.5 km SSW Guanaceví (Mina 6) | 25.9 | -106 | 24/07/2001 |
| CRD3182 | M | Durango | SMOC | 3 km N, 3.5 km W Guanaceví (Mina 25) | 25.96 | -106 | 26/07/2001 |
| CRD3183 | F | Durango | SMOC | 3 km N, 3.5 km W Guanaceví (Mina 25) | 25.96 | -106 | 26/07/2001 |
| CRD3184 | F | Durango | SMOC | 3 km N, 3.5 km W Guanaceví (Mina 25) | 25.96 | -106 | 26/07/2001 |
| CRD3185 | F | Durango | SMOC | 3 km N, 3.5 km W Guanaceví (Mina 25) | 25.96 | -106 | 26/07/2001 |
| CRD3186 | M | Durango | SMOC | 3 km N, 3.5 km W Guanaceví (Mina 25) | 25.96 | -106 | 26/07/2001 |
| CRD3187 | M | Durango | SMOC | 3 km N, 3.5 km W Guanaceví (Mina 25) | 25.96 | -106 | 26/07/2001 |
| CRD3188 | M | Durango | SMOC | 3 km N, 3.5 km W Guanaceví (Mina 25) | 25.96 | -106 | 26/07/2001 |
| CRD3189 | F | Durango | SMOC | 3 km N, 3.5 km W Guanaceví (Mina 25) | 25.96 | -106 | 26/07/2001 |
| CRD3190 | F | Durango | SMOC | 3 km N, 3.5 km W Guanaceví (Mina 25) | 25.96 | -106 | 26/07/2001 |
| CRD3197 | M | Durango | SMOC | 2 km WSW Guanaceví (Mina 4) | 25.92 | -106 | 28/07/2001 |
| CRD3293 | M | Durango | SMOC | 1 km S Guanaceví | 25.92 | -106 | 08/01/2002 |
| CRD3295 | F | Durango | SMOC | Mina "La Mexicana" 3.5 km SSW Guanaceví (Mina 6) | 25.9 | -106 | 08/01/2002 |
| CRD3297 | F | Durango | SMOC | Mina "La Mexicana" 3.5 km SSW Guanaceví (Mina 6) | 25.9 | -106 | 08/01/2002 |
| CRD3298 | M | Durango | SMOC | Mina "La Mexicana" 3.5 km SSW Guanaceví (Mina 6) | 25.9 | -106 | 08/01/2002 |
| CRD3300 | M | Durango | SMOC | 3 km N, 3.5 km W Guanaceví (Mina 24) | 25.96 | -106 | 10/01/2002 |
| CRD3301 | F | Durango | SMOC | 3 km N, 3.5 km W Guanaceví (Mina 24) | 25.96 | -106 | 10/01/2002 |
| CRD3302 | F | Durango | SMOC | 3 km N, 3.5 km W Guanaceví (Mina 24) | 25.96 | -106 | 10/01/2002 |
| CRD3303 | F | Durango | SMOC | 3 km N, 3.5 km W Guanaceví (Mina 24) | 25.96 | -106 | 10/01/2002 |
| CRD3304 | F | Chihuahua | SMOC | 3 km N, 3.5 km W Guanaceví (Mina 24) | 25.96 | -106 | 10/01/2002 |
| CRD3305 | F | Durango | SMOC | 3.1 km N, 3.5 km W Guanaceví (Mina 22) | 25.96 | -106 | 11/01/2002 |
| CRD3306 | F | Durango | SMOC | 1.75 km NW Guanaceví | 25.94 | -106 | 09/01/2002 |
| CRD4829 | M | Chihuahua | SMOC | Mina Piedras Verdes, 0.5 km W San Antonio | 27.09 | -108 | 23/03/2005 |
| CRD4830 | F | Durango | SMOC | Cerro Blanco | 23.47 | -104 | 21/06/2004 |
| CRD5079 | F | Durango | SMOC | Mina "La Mexicana" 3.5 km SSW Guanaceví (Mina 6) | 25.9 | -106 | 28/01/2006 |
| CRD5080 | F | Durango | SMOC | Mina "La Mexicana" 3.5 km SSW Guanaceví (Mina 6) | 25.9 | -106 | 28/01/2006 |
| CRD5469 | M | Durango | SMOC | 0.3 km S, 2.2 km E Guanaceví | 25.93 | -106 | 21/05/2008 |
| CRD11772 | F | Nuevo León | SMO | 7.5 km N, 6 km W San Josecito | 24.04 | -100 | 13/07/1990 |
| CRD11773 | F | Nuevo León | SMO | Cueva de San Josecito, 1.3 km S, 0.8 km W San Josecito | 23.96 | -99.9 | 27/12/1988 |
| CRD11774 | M | Nuevo León | SMO | Cueva de San Josecito, 1.3 km S, 0.8 km W San Josecito | 23.96 | -99.9 | 27/12/1988 |
| CRD11775 | M | Nuevo León | SMO | Cueva de San Josecito, 1.3 km S, 0.8 km W San Josecito | 23.96 | -99.9 | 27/12/1988 |
| CRD11776 | M | Nuevo León | SMO | Cueva de San Josecito, 1.3 km S, 0.8 km W San Josecito | 23.96 | -99.9 | 27/12/1988 |
| CRD11777 | M | Nuevo León | SMO | Cueva de San Josecito, 1.3 km S, 0.8 km W San Josecito | 23.96 | -99.9 | 27/12/1988 |
| CRD11778 | F | Nuevo León | SMO | Cueva de San Josecito, 1.3 km S, 0.8 km W San Josecito | 23.96 | -99.9 | 19/03/1989 |
| CRD11779 | M | Nuevo León | SMO | Cueva de San Josecito, 1.3 km S, 0.8 km W San Josecito | 23.96 | -99.9 | 19/03/1989 |
| CNMA18612 | M | Ciudad de México | TMVB | Pedregal de Guadalupe Hidalgo | 19.25 | -99.5 | 23/05/1981 |
| CNMA19693 | M | Ciudad de México | TMVB | Cañada de La Toma, 0.5 km SW Temascalcingo | 19.91 | -100 | 28/08/1982 |
| CNMA19694 | M | México | TMVB | 2 km W El Oro | 19.8 | -100 | 29/08/1982 |
| CNMA18529 | M | Michoacán | TMVB | Albergue Los Azufres | 19.78 | -101 | 16/11/1979 |
| CNMA18530 | M | Michoacán | TMVB | Albergue Los Azufres | 19.78 | -101 | 16/11/1979 |
| CNMA33927 | M | Michoacán | TMVB | 6 km NW Playa Azul | 18 | -102 | 23/03/1979 |
| CNMA7163 | M | Morelos | TMVB | Cerro El Fraile, Cueva del Murciélago, 6.5 km NW Tres Marías | 19.1 | -99.3 | 07/10/1962 |
| CNMA20092 | F | Querétaro | TMVB | 14 km N Jalpan | 21.34 | -99.5 | 05/07/1983 |
| CNMA22481 | F | San Luis Potosí | SMOC | Cueva La Joya | 21.99 | -100 | 19/07/1983 |
| CNMA34811 | M | Tamaulipas | SMO | Cueva del Charco de la Perra, 11.6 km NW Gómez Farías | 23.12 | -99.2 | 18/10/1991 |
| CNMA34812 | F | Tamaulipas | SMO | Cueva del Charco de la Perra, 11.6 km NW Gómez Farías | 23.12 | -99.2 | 15/12/1991 |
| CNMA34813 | M | Tamaulipas | SMO | Cueva del Charco de la Perra, 11.6 km NW Gómez Farías | 23.12 | -99.2 | 11/03/1991 |
| CNMA34814 | F | Tamaulipas | SMO | Cueva del Charco de la Perra, 11.6 km NW Gómez Farías | 23.12 | -99.2 | 11/03/1991 |
| CNMA34815 | F | Tamaulipas | SMO | Cueva del Charco de la Perra, 11.6 km NW Gómez Farías | 23.12 | -99.2 | 11/03/1991 |
| CNMA34817 | F | Tamaulipas | SMO | Cueva La Capilla, 11.5 km NW Gómez Farías | 23.12 | -99.2 | 13/05/1992 |
| CNMA34818 | F | Tamaulipas | SMO | Cueva La Capilla, 11.5 km NW Gómez Farías | 23.12 | -99.2 | 12/06/1992 |
| MZFC-M3647 | F | Querétaro | TMVB | San Joaquín, 6 Km SSE | 20.87 | -99.5 | 19/03/1984 |
| MZFC-M3648 | M | Querétaro | TMVB | San Joaquín, 6 Km SSE | 20.87 | -99.5 | 19/03/1984 |
| MZFC-M4064 | F | Querétaro | TMVB | Pinal de Amoles, Cerro del Judío | 21.13 | -99.6 | 28/11/1986 |
| MZFC-M9207 | M | Querétaro | TMVB | Poblado de Chavarría | 20.81 | -99.6 | 08/06/1998 |
| MZFC-M9208 | M | Querétaro | TMVB | Poblado de Chavarría | 20.81 | -99.6 | 08/06/1998 |
| MZFC-M10780 | M | Ciudad de México | TMVB | MC8 SD | 19.28 | -99.3 | 19/03/2007 |
| MZFC-M10782 | M | Ciudad de México | TMVB | MC8 SD | 19.28 | -99.3 | 19/03/2007 |
| MZFC-M11498 | F | Michoacán | TMVB | Parque Nacional Barranca del Cupatitzio | 19.43 | -102 | 13/06/2010 |
| MZFC-M12347 | M | Ciudad de México | TMVB | Magdalena Contreras | 19.28 | -99.3 | 13/11/2007 |
| MZFC-M12845 | F | Ciudad de México | TMVB | San Pablo Oztotepec | 19.11 | -99.1 | 05/12/2008 |
| MZFC-M13050 | M | Puebla | TMVB | Cerro Las Espejeras, Interior de Mina | 19.78 | -97.8 | 17/11/2012 |
| MZFC-M13051 | F | Puebla | TMVB | Cerro Las Espejeras, Interior de Mina | 19.78 | -97.8 | 17/11/2012 |
| ENCB3807 | M | Veracruz | TMVB | 10 km SE Perote | 19.5 | -97.2 | 03/05/1968 |
| ENCB3808 | M | Veracruz | TMVB | 10 km SE Perote | 19.5 | -97.2 | 03/05/1968 |
| ENCB3810 | M | Veracruz | TMVB | 10 km SE Perote | 19.5 | -97.2 | 03/05/1968 |
| ENCB4396 | M | Tlaxcala | TMVB | 13 km S Calpulalpan | 19.48 | -98.6 | 21/02/1970 |
| ENCB4402 | M | Tlaxcala | TMVB | 13 km S Calpulalpan | 19.48 | -98.6 | 21/02/1970 |
| ENCB4404 | M | Tlaxcala | TMVB | 13 km S Calpulalpan | 19.48 | -98.6 | 21/02/1970 |
| ENCB4405 | M | Tlaxcala | TMVB | 13 km S Calpulalpan | 19.48 | -98.6 | 21/02/1970 |
| ENCB4410 | M | Tlaxcala | TMVB | 13 km S Calpulalpan | 19.48 | -98.6 | 21/02/1970 |
| ENCB26561 | F | Michoacán | TMVB | 5 Km S, 1 Km W Dos Aguas | 18.76 | -103 | 21/10/1986 |
| ENCB26562 | F | Michoacán | TMVB | 5 Km S, 1 Km W Dos Aguas | 18.76 | -103 | 21/10/1986 |
| ENCB26563 | M | Michoacán | TMVB | 5 Km S, 1 Km W Dos Aguas | 18.76 | -103 | 21/10/1986 |
| ENCB26564 | F | Michoacán | TMVB | 5 Km S, 1 Km W Dos Aguas | 18.76 | -103 | 21/10/1986 |
| ENCB26565 | F | Michoacán | TMVB | 5 Km S, 1 Km W Dos Aguas | 18.76 | -103 | 21/10/1986 |
| ENCB26566 | F | Michoacán | TMVB | 5 Km S, 1 Km W Dos Aguas | 18.76 | -103 | 21/10/1986 |
| ENCB26567 | F | Michoacán | TMVB | 5 Km S, 1 Km W Dos Aguas | 18.76 | -103 | 21/10/1986 |
| ENCB26568 | F | Michoacán | TMVB | 5 Km S, 1 Km W Dos Aguas | 18.76 | -103 | 21/10/1986 |
| ENCB26569 | F | Michoacán | TMVB | 5 Km S, 1 Km W Dos Aguas | 18.76 | -103 | 21/10/1986 |
| ENCB27391 | M | Querétaro | TMVB | Pinal de Amoles | 21.13 | -99.6 | 31/01/1987 |
| ENCB27986 | M | Puebla | TMVB | Ciudad Serdán; 1.5 km NE | 19 | -97.4 | 24/03/1987 |
| ENCB27987 | M | Puebla | TMVB | Ciudad Serdán; 1 km SW | 18.98 | -97.5 | 25/03/1987 |
| ENCB27988 | M | Puebla | TMVB | Ciudad Serdán; 1 km SW | 18.98 | -97.5 | 25/03/1987 |
| ENCB29365 | M | Jalisco | TMVB | 2 Km S, 6 Km El Jazmín | 19.63 | -104 | 10/04/1988 |
| ENCB32073 | M | San Luis Potosí | SMOC | 3 Km SE Álvarez | 22.01 | -101 | 12/04/1989 |
| ENCB32074 | F | San Luis Potosí | SMOC | 3 Km SE Álvarez | 22.01 | -101 | 12/04/1989 |
| ENCB32075 | M | San Luis Potosí | SMOC | 3 Km SE Álvarez | 22.01 | -101 | 12/04/1989 |
| ENCB41589 | F | Ciudad de México | TMVB | La Quinta, 11 Km S, 2 Km E Santa Ana Tlacotenco, Milpa Alta | 19.08 | -99 | 05/05/2000 |
| ENCB41590 | F | Ciudad de México | TMVB | La Quinta, 11 Km S, 2 Km E Santa Ana Tlacotenco, Milpa Alta | 19.08 | -99 | 05/05/2000 |
| ENCB41591 | F | Ciudad de México | TMVB | La Quinta, 11 Km S, 2 Km E Santa Ana Tlacotenco, Milpa Alta | 19.08 | -99 | 05/05/2000 |
| ENCB41592 | F | Ciudad de México | TMVB | La Quinta, 11 Km S, 2 Km E Santa Ana Tlacotenco, Milpa Alta | 19.08 | -99 | 05/05/2000 |
| ENCB42211 | M | Ciudad de México | TMVB | La Quinta, 11 Km S, 2 Km E Santa Ana Tlacotenco, Milpa Alta | 19.08 | -99 | 05/05/2001 |
| ENCB42212 | M | Ciudad de México | TMVB | La Quinta, 11 Km S, 2 Km E Santa Ana Tlacotenco, Milpa Alta | 19.08 | -99 | 05/05/2001 |
| ENCB42213 | F | Ciudad de México | TMVB | La Quinta, 11 Km S, 2 Km E Santa Ana Tlacotenco, Milpa Alta | 19.08 | -99 | 05/05/2001 |
| ENCB42214 | F | Ciudad de México | TMVB | La Quinta, 11 Km S, 2 Km E Santa Ana Tlacotenco, Milpa Alta | 19.08 | -99 | 05/05/2001 |
| ENCB42215 | F | Ciudad de México | TMVB | La Quinta, 11 Km S, 2 Km E Santa Ana Tlacotenco, Milpa Alta | 19.08 | -99 | 05/05/2001 |
| ENCB42216 | F | Ciudad de México | TMVB | La Quinta, 11 Km S, 2 Km E Santa Ana Tlacotenco, Milpa Alta | 19.08 | -99 | 05/05/2001 |
| ENCB43631 | F | Hidalgo | TMVB | San Rafael Amolucan | 19.98 | -98.5 | 09/10/2004 |
| ENCB43632 | F | Hidalgo | TMVB | San Rafael Amolucan | 19.98 | -98.5 | 09/10/2004 |
| UAM-I2742 | M | Hidalgo | TMVB | 8 Km E Singuilucan | 19.96 | -98.5 | 25/03/1980 |
| UAM-I2743 | M | Hidalgo | TMVB | 8 Km E Singuilucan | 19.96 | -98.5 | 25/03/1980 |
| UAM-I15265 | M | Michoacán | TMVB | Chiqueritos, 1.8 Km N Dos Aguas | 18.82 | -103 | 31/01/1995 |
| UAM-I15266 | M | Michoacán | TMVB | Chiqueritos, 1.8 Km N Dos Aguas | 18.82 | -103 | 31/01/1995 |
| UAM-I15267 | M | Michoacán | TMVB | Chiqueritos, 1.8 Km N Dos Aguas | 18.82 | -103 | 31/01/1995 |
| UAM-I15268 | M | Michoacán | TMVB | 1 km NE Dos Aguas | 18.8 | -103 | 03/02/1995 |
| UAM-I15269 | M | Michoacán | TMVB | 1 km NE Dos Aguas | 18.8 | -103 | 03/02/1995 |
| UAM-I15270 | M | Michoacán | TMVB | 1 km NE Dos Aguas | 18.8 | -103 | 03/02/1995 |
| UAM-I15271 | M | Michoacán | TMVB | 1 km NE Dos Aguas | 18.8 | -103 | 03/02/1995 |
| UAM-I15272 | M | Michoacán | TMVB | 1 km NE Dos Aguas | 18.8 | -103 | 03/02/1995 |
| UAM-I15273 | F | Michoacán | TMVB | 1 km NE Dos Aguas | 18.8 | -103 | 03/02/1995 |
| UAM-I15274 | M | Michoacán | TMVB | 1 km NE Dos Aguas | 18.8 | -103 | 03/02/1995 |
| UAM-I15275 | M | Michoacán | TMVB | 1 km NE Dos Aguas | 18.8 | -103 | 03/02/1995 |
| UAM-I15276 | M | Michoacán | TMVB | La Nieve, 12 Km E Dos Aguas | 18.83 | -103 | 07/09/1997 |
| UAM-I15277 | M | Michoacán | TMVB | La Nieve, 12 Km E Dos Aguas | 18.83 | -103 | 07/09/1997 |
| UAM-I15278 | M | Michoacán | TMVB | La Nieve, 12 Km E Dos Aguas | 18.83 | -103 | 07/09/1997 |
| UAM-I15280 | M | Michoacán | TMVB | Cerro el Ventero, 1 Km NE Presa Pucuato | 19.63 | -101 | 02/11/1994 |
| IIB1030 | M | Veracruz | TMVB | Plan de Sedeño, 5 Km N | - | - | 01/06/1989 |
| IIB1031 | M | Veracruz | TMVB | Plan de Sedeño, 5 Km N | - | - | 01/06/1989 |
| IIB1979 | M | Veracruz | TMVB | Plan de Sedeño, 5 Km N | - | - | 16/01/1993 |
| IIB3121 | F | Veracruz | TMVB | Pueblo Nuevo-Las minas | - | - | 20/05/2006 |
| IIB517 | M | Veracruz | TMVB | Volcancillo | - | - | 24/06/1987 |
| IIB1032 | F | Veracruz | TMVB | Plan de Sedeño, 5 Km N | - | - | 01/06/1989 |
| UANL242 | F | Nuevo León | SMO | Cueva La Joya Honda, 1 km NE General Zaragoza. | 23.97 | -99.8 | 24/03/1967 |
| UANL1661 | F | Nuevo León | SMO | Cueva La Joya Honda, 1 km NE General Zaragoza. | 23.97 | -99.8 | 20/03/1971 |
| UANL1664 | F | Nuevo León | SMO | Cueva La Cuesta Blanca, 2.5 km N General Zaragoza. | 23.97 | -99.8 | 20/03/1971 |
| UANL1667 | F | Nuevo León | SMO | Colonia Coutry, La Silla, Monterrey | 25.69 | -100 | 21/05/1973 |
| UANL2622 | F | Nuevo León | SMO | Cueva El Infierno, La Camotera | 25.12 | -100 | NA/NA/2018 |
| SLPZ0006 | M | San Luis Potosí | SMOC | Sierra de Álvarez | 22.06 | -101 | 20/07/2018 |
| SLPZ0050 | F | San Luis Potosí | SMOC | Sierra de Álvarez | 22.08 | -101 | 08/07/2019 |
| SLPZ0031 | F | San Luis Potosí | SMOC | Sierra de Álvarez | 22.1 | -101 | 05/05/2019 |
| SLPZ0032 | M | San Luis Potosí | SMOC | Sierra de Álvarez | 22.1 | -101 | 05/05/2019 |
| SLPZ0043 | M | San Luis Potosí | SMOC | Sierra de Álvarez | 22.08 | -101 | 01/07/2019 |
| UAZ1 | - | Zacatecas | SMOC | Susticacán | 22.61 | -103 | - |
| UAEH-CIB2170 | F | Hidalgo | TMVB | Villa Juárez, PN Los Mármoles | 20.85 | -99.2 | 26/10/2017 |
| UAEH-CIB2367 | M | Hidalgo | TMVB | Jaguey Colorado, PN Los Mármoles | 20.9 | -99.1 | 06/09/2018 |
| UAEH-CIB2171 | M | Hidalgo | TMVB | Villa Juárez, PN Los Mármoles | 20.85 | -99.2 | 05/11/2017 |
| UAEH-CIB2175 | F | Hidalgo | TMVB | Jaguey Colorado, PN Los Mármoles | 20.88 | -99.3 | 06/10/2017 |
| UAEH-CIB2359 | M | Hidalgo | TMVB | Jaguey Colorado, PN Los Mármoles | 20.9 | -99.1 | 13/08/2018 |
| UAEH-CIB2360 | F | Hidalgo | TMVB | Jaguey Colorado, PN Los Mármoles | 20.87 | -99.3 | 13/08/2018 |
| UAEH-CIB2361 | F | Hidalgo | TMVB | Jaguey Colorado, PN Los Mármoles | 20.87 | -99.3 | 13/08/2018 |
