# Supplementary material for: Molecular and morphological data suggest a new species of big-eared bat (Vespertilionidae: Corynorhinus) endemic to northeastern Mexico
Source: PLoS One. 2024 Feb 21;19(2):e0296275. doi: 10.1371/journal.pone.0296275 (PMC10881012; doi:10.1371/journal.pone.0296275)
Supplement: S2 Appendix — (DOCX) [file pone.0296275.s002.docx]

**Molecular and morphological data suggest a new species of big-eared bat (Vespertilionidae: *Corynorhinus*) endemic to northeastern Mexico**

**S2 Appendix**

| **Table A. Primer sequences used for amplifying cytochrome c oxidase subunit I (COI) and cytochrome b (Cyt-b) mitochondrial genes and the recombination activating gene 2 (RAG2) nuclear gene.** | | | | |
| --- | --- | --- | --- | --- |
| **Gene** | **Code** | **Primer** | **Length** | **Reference** |
| COI | F | 5’- CCTGCTAATCCGTGCTGAAC -3’ | ~200 pb | deNovo |
|  | R | 5’- AGAGGGTGGAAGGAGTCAGA -3’ |  |  |
| Cyt-b | HCB | 5’ -TACATAGACTCTTCATTT-3’ | 872 pb | Piaggio and Perkins, 2005 |
|  | L2 | 5’-GCCAACGGRGCCTCYATA-3’ |  |  |
| RAG2 | RAG2F220 | 5’- GATTCCTGCTAYCTYCCTCCTCT -3’ | 766 pb | Teeling et al., 2000 |
|  | RAG2R995 | 5’- CCCATGTTGCTTCCAAACCATA -3’ |  |  |

| **Table B. Detail of time and temperature protocols used in the amplification of the DNA fragments.** | | | | | | |
| --- | --- | --- | --- | --- | --- | --- |
| **Gene** | **Denature** | **Cycles** | **Denature** | **Annealing** | **Extension** | **Final extension** |
| Cyt-b | 94°C - 4 min | 35 | 94°C - 30 s | 47°C - 30s | 72°C - 1 min | 72°C - 7 min |
| COI | 95° - 5 min | 35 | 95° - 1min | 52° - 1 min | 72° - 1 min | 72° - 10 min |
| RAG2 | 95°C - 3 min | 35 | 95°C - 30 s | 58°C - 1min | 72°C - 2 min | 72°C - 5 min |

| **Table C. Summary of individuals of *Corynorhinus mexicanus* used in the three previous time divergence analyses (see methods). Certain individuals were included more than once due to their unique representation of the locality. For outgroups (*Corynorhinus townsendii* and *Plecotus auritus*), we used the same sequences of Cyt-*b* in the three analyses. GenBank accession numbers of the outgroup sequences are shown. Abbreviations: SMO, Sierra Madre Oriental; SMOC, Sierra Madre Occidental; TMVB, Trans-Mexican Volcanic Belt.** | | | |
| --- | --- | --- | --- |
| **Species/linage** | **Analysis 1** | **Analysis 2** | **Analysis 3** |
| *C. mexicanus* SMOC | Cm58ZAC | Cm58ZAC | Cm58ZAC |
|  | Cm2SLP | Cm5SLP | Cm3SLP |
|  | Cm11DUR | Cm14DUR | Cm21DUR |
| *C. mexicanus* SMO | Cm8NL | Cm55NL | Cm7NL |
| *C. mexicanus* TMVB | Cm57TLX | Cm30TLX | Cm29TLX |
|  | Cm35VER | Cm38VER | Cm43VER |
|  | Cm56JAL | Cm56JAL | Cm56JAL |
|  | Cm54QRO | Cm54QRO | Cm54QRO |
|  | Cm3HGO | Cm5HGO | Cm8HGO |
| *C. townsendii* | Ct10HGO | Ct59ZAC | Ct32MOR |
| *C. rafinesquii* | NC_016872.1 | NC_016872.1 | NC_016872.1 |
| *Plecotus auritus* | AB085734.1 | AB085734.1 | AB085734.1 |


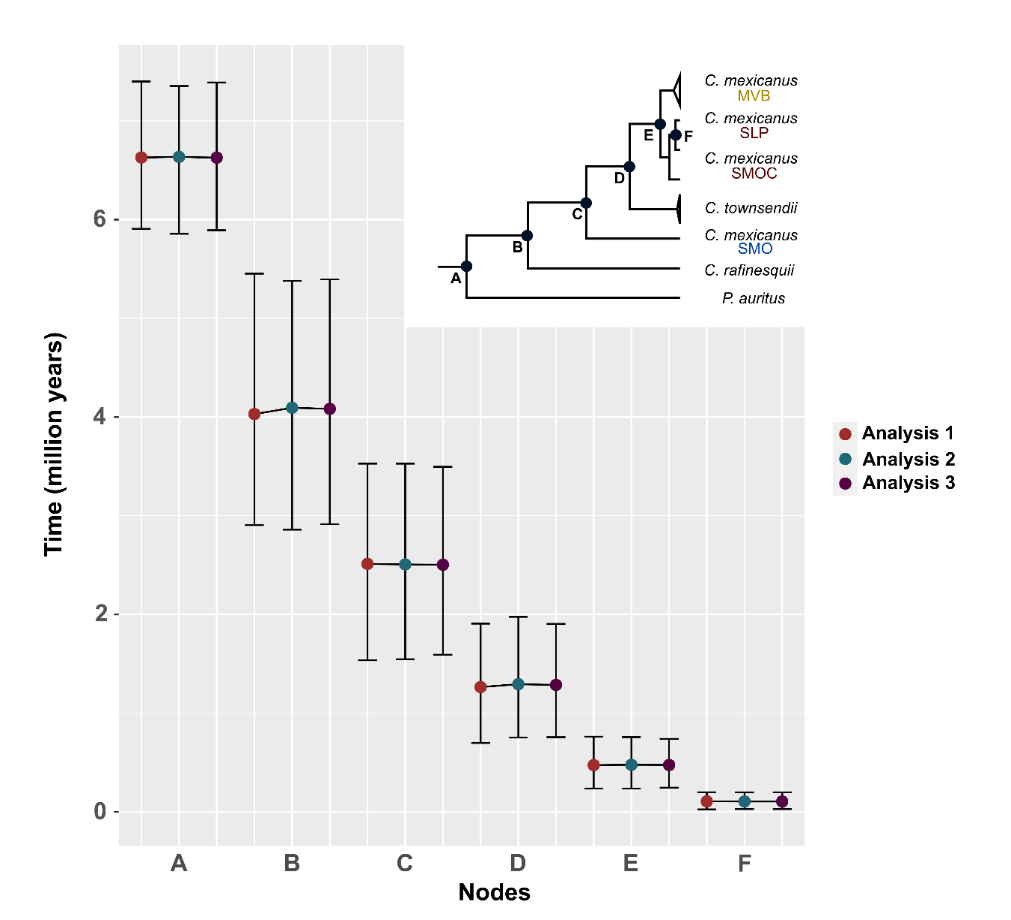


**Fig A.** Comparison of mean and 95% Highest Posterior Density values of common ancestor height estimated during three previous time divergence analyses (see methods). Node identity is shown in the phylogeny (upper right). Abbreviations: SMO, Sierra Madre Oriental; SMOC, Sierra Madre Occidental; TMVB, Trans-Mexican Volcanic Belt.


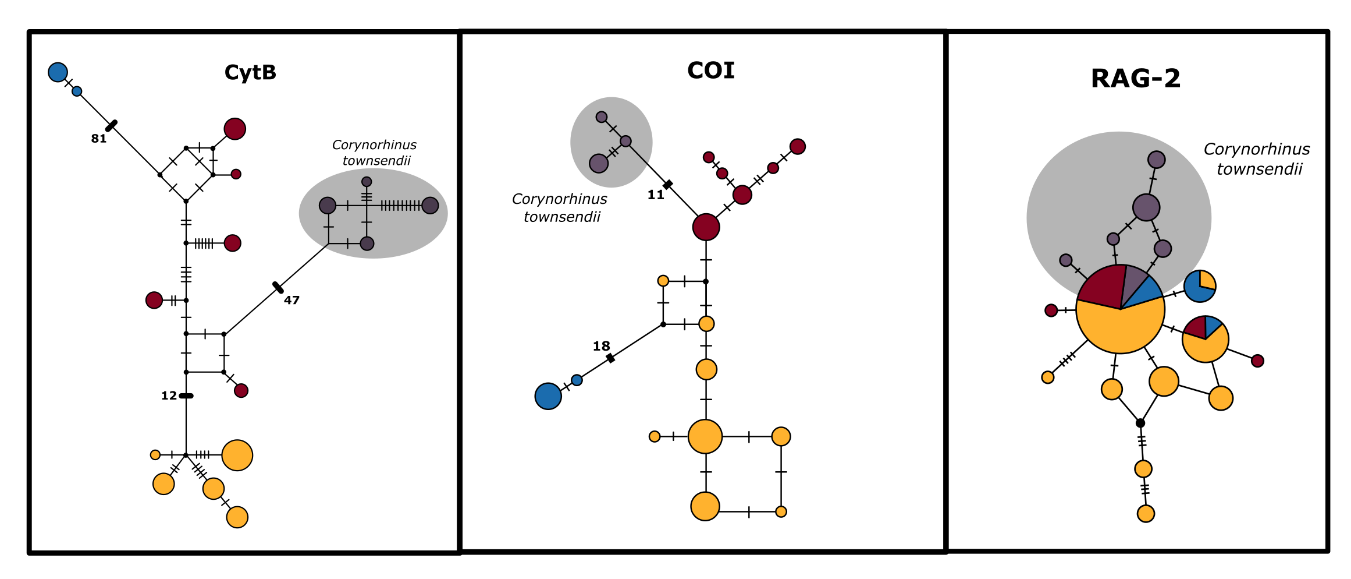


**Fig B.** Network of haplotypes of Cyt-*b*, COI, and RAG2. The size of the circles is proportional to the number of samples present in the haplotype, and black lines indicate observed mutational steps. Color codes correspond to haplogroups found in the mitochondrial concatenate haplotype network. Haplogroups of SMO are shown in blue, SMOC in red, and TMVB in yellow, whereas *C. townsendii* haplogroup is shown in gray.


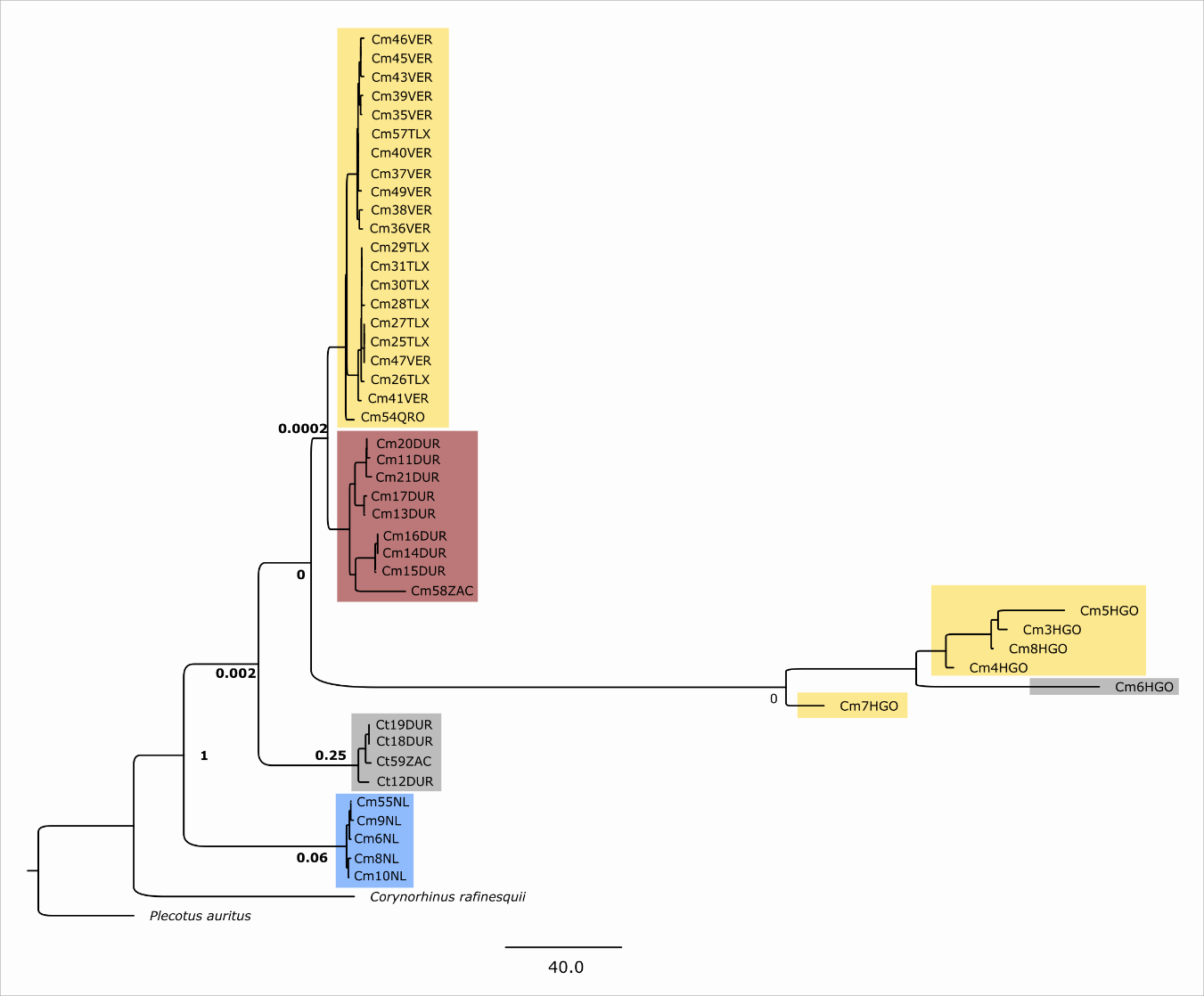


**Fig C.** Tree which summarized the NJ LILD results using COI, Cyt-*b*, and RAG2. Branches affected by incongruence are indicated by values in branches (*p* < 0.05). *P* values of terminal branches are not shown. Individuals from SMO are shown in blue, from TMVB in yellow, and from SMOC in red, whereas *C. townsendii* is shown in gray.


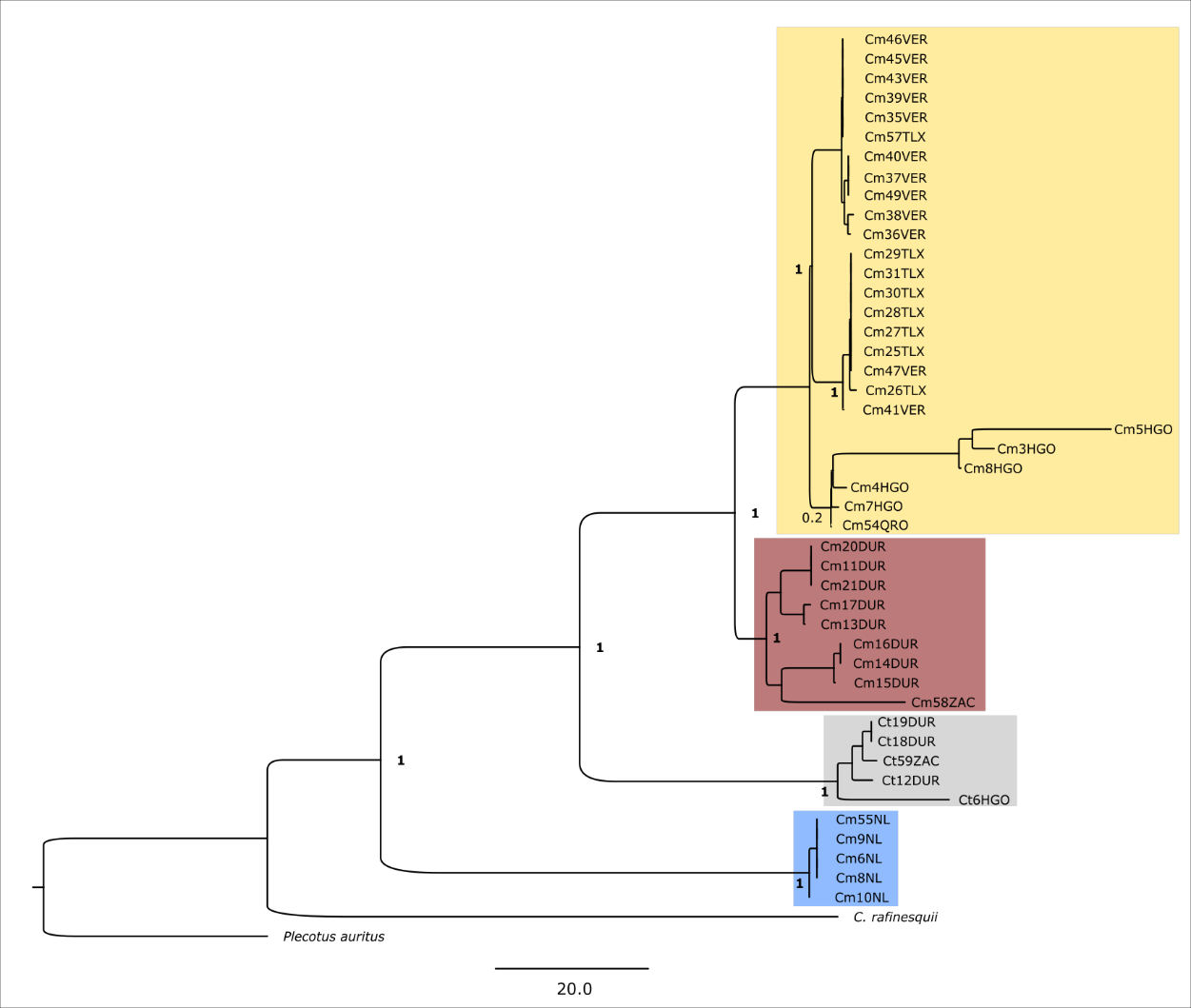


**Fig D.** Tree which summarized the NJ LILD results using COI and Cyt-*b*. Branches affected by incongruence are indicated by values in branches (*p* < 0.05). *P* values of terminal branches are not shown. Individuals from SMO are shown in blue, from TMVB in yellow, and from SMOC in red. whereas *C. townsendii* is shown in gray.
