# Supplementary material for: Molecular and morphological data suggest a new species of big-eared bat (Vespertilionidae: Corynorhinus) endemic to northeastern Mexico
Source: PLoS One. 2024 Feb 21;19(2):e0296275. doi: 10.1371/journal.pone.0296275 (PMC10881012; doi:10.1371/journal.pone.0296275)
Supplement: S3 Appendix — (DOCX) [file pone.0296275.s003.docx]

**Molecular and morphological data suggest a new species of big-eared bat (Vespertilionidae: *Corynorhinus*) endemic to northeastern Mexico**

**S3 Appendix**

**Sequencing and assembling of mitogenomes.**

Three individuals of *C. mexicanus* captured in the field were euthanized using isoflurane, a volatile anesthetic that causes no specific signs of distress or pain in individuals (see methods). Two individuals were collected in Puerto Grade, Galeana in the state of Nuevo León, Mexico, and one individual was collected in La Malinche National Park in Tlaxcala, Mexico. The localities are located in Sierra Madre Oriental (SMO) and Transmexican Volcanic Belt (TMVB), respectively. After individuals were euthanized, we dissected them and collected tissue samples from the liver, pectoral muscle, heart, and lung. These tissues were stored in 96% ethanol and transported to the laboratory for further processing. For DNA extraction, we used tissues samples of the liver and pectoral muscle. The remaining tissues samples were deposited in the Colección de Tejidos de Vertebrados of the Escuela Nacional de Ciencias Biológicas, ENCB-IPN (#catalogue: ENCB_Chis-Ves_0041) and Colección de Mamíferos del Museo de Zoología “Alfonso L. Herrera”, FC-UNAM, (#catalogue: MZFC-M16326).

Ethanol was removed from tissue using 45 ml of buffer solution composed of 400 mM NaCl, 20 mM Tris-HCl with pH 7.5, and 30 mM EDTA. Samples were shaken over 30 minutes at room temperature and dried with Kimwipe towels. The process was repeated twice.

The ethanol-free tissue samples were put in sterile 1.5 ml tubes. When ready to extract DNA, 1 ml of phosphate buffered saline (PBS) was added and tissues were crushed in small fragments with sterile scissors. Tissue-PBS solution was centrifuged for 10 minutes at 10,000 x g. The supernatant was discarded. We added 200 µl of Lysis Buffer Solution Zymo Research® and 20 µl of Proteinase K to the 1.5 ml tubes with tissue fragments. We incubated the solution for 24 hours at 55°C with slight shaking. Finally, the solution was centrifuged to 10,000 x g for 1 minute. The supernatant was saved, and solid residues were discarded.

The extraction of DNA varies among tissue samples. For samples of the SMOR lineage, we used the commercial kit EZ10-Spin Column Animal Genomic DNA© (Gentech Biosciences ®) following the manufacturer's instructions. For samples of the SMOC-TMVB linage, the supernatant recovered during the lysis phase was put in a novel 1.5 ml sterile tube. We added 10 µl of RNase to the supernatant solution and incubate it for 5 minutes. Next, we added 150 µl of magnetic beads and Buffer W of Qiagen©. The solution was incubated for 1 hour and centrifuged to 10,000 x g for 1 minute. Tubes with the solution were put over magnetic support and the solution was reposed for 10 minutes. After that, the supernatant was discarded. We added 150 ml of ethanol 80% avoiding moving the tubes or magnetic beads. Ethanol was discarded by decantation. We repeated the ethanol washing step three times. Finally, DNA was obtained from the magnetic beads using 100 µl of EB solution of Qiagen©. The DNA solution was put in a 1.5 ml tube and stored at 4°C.

Sequencing was done by Novogene® (Sacramento, California) using Illumina synthesis technology in the Novaseq 6000 equipment and an S4 flow cell with a sequence length of 150 bp.

**Table A.** Models and partitions used for phylogenetic analysis using entirely mitochondrial genomes. The length of each partition indicates the position of each element in an aligned sequences matrix of 14930 bp. The suffixes 1^st^, 2^nd^, and 3^rd^ in name partitions indicate the codon position of the protein coding genes.

| **Partition** | **Substitution model** | **Length** |
| --- | --- | --- |
| Ribosomal RNA | TN+Γ | 70-1035, 1106-2672 |
| Transfer RNA | HKY+Γ | 1-69, 1036-1105, 2673-2748, 3710-3777, 3849-3917, 4959-5026, 5032-5100, 5102-5174, 5175-5209, 5207-5272, 5273-5345, 6898-6966, 6974-7040, 7729-7794, 9421-9490, 9838-9907, 11579-11647, 11648-11708, 11709-11779, 13583-13655, 14796-14865, 14865-14930 |
| nad1 1st | TN+I | 2754-3709\3 |
| nad1 2nd | HKY+I | 2755-3709\3 |
| nad1 3rd | HKY | 2756-3709\3 |
| nad2 1st | HKY+I | 3918-4959\3 |
| nad2 2nd | HKY+I | 3919-4959\3 |
| nad2 3rd | HKY | 3920-4959\3 |
| cox1 1st | TN+I | 5345-6889\3 |
| cox1 2nd | TN | 5346-6889\3 |
| cox1 3rd | TN+I | 5347-6889\3 |
| cox2 1st | TN | 7041-7724\3 |
| cox2 2nd | HKY | 7042-7724\3 |
| cox2 3rd | HKY | 7043-7724\3 |
| atp8 1st | HKY+Γ | 7796-7999\3 |
| atp8 2nd | HKY | 7797-7999\3 |
| atp8 3rd | TN+Γ | 7798-7999\3 |
| atp6 1st | TN+I | 7957-8637\3 |
| atp6 2nd | HKY | 7958-8637\3 |
| atp6 3rd | HKY | 7959-8637\3 |
| cox3 1st | HKY+I | 8637-9420\3 |
| cox3 2nd | HKY | 8638-9420\3 |
| cox3 3rd | HKY+Γ | 8639-9420\3 |
| nad3 1st | HKY+I | 9491-9836\3 |
| nad3 2nd | HKY | 9492-9836\3 |
| nad3 3rd | HKY+Γ | 9493-9836\3 |
| nad4L 1st | HKY | 9909-10207\3 |
| nad4L 2nd | HKY | 9910-10207\3 |
| nad4L 3rd | TN+I | 9911-10207\3 |
| nad4 1st | TN+Γ | 10201-11578\3 |
| nad4 2nd | HKY+I | 10202-11578\3 |
| nad4 3rd | HKY+I | 10203-11578\3 |
| nad5 1st | TN+I | 11780-13599\3 |
| nad5 2nd | TN+Γ | 11781-13599\3 |
| nad5 3rd | HKY+Γ | 11782-13599\3 |
| cyt-b 1st | TN+I | 13656-14795\3 |
| cyt-b 2nd | HKY+I | 13657-14795\3 |
| cyt-b 3rd | HKY+I | 13658-14795\3 |
