# Supplementary material for: Molecular and morphological data suggest a new species of big-eared bat (Vespertilionidae: Corynorhinus) endemic to northeastern Mexico
Source: PLoS One. 2024 Feb 21;19(2):e0296275. doi: 10.1371/journal.pone.0296275 (PMC10881012; doi:10.1371/journal.pone.0296275)
Supplement: S4 Appendix — (DOCX) [file pone.0296275.s004.docx]

**Molecular and morphological data suggest a new species of big-eared bat (Vespertilionidae: *Corynorhinus*) endemic to northeastern Mexico**

**S4 Appendix**


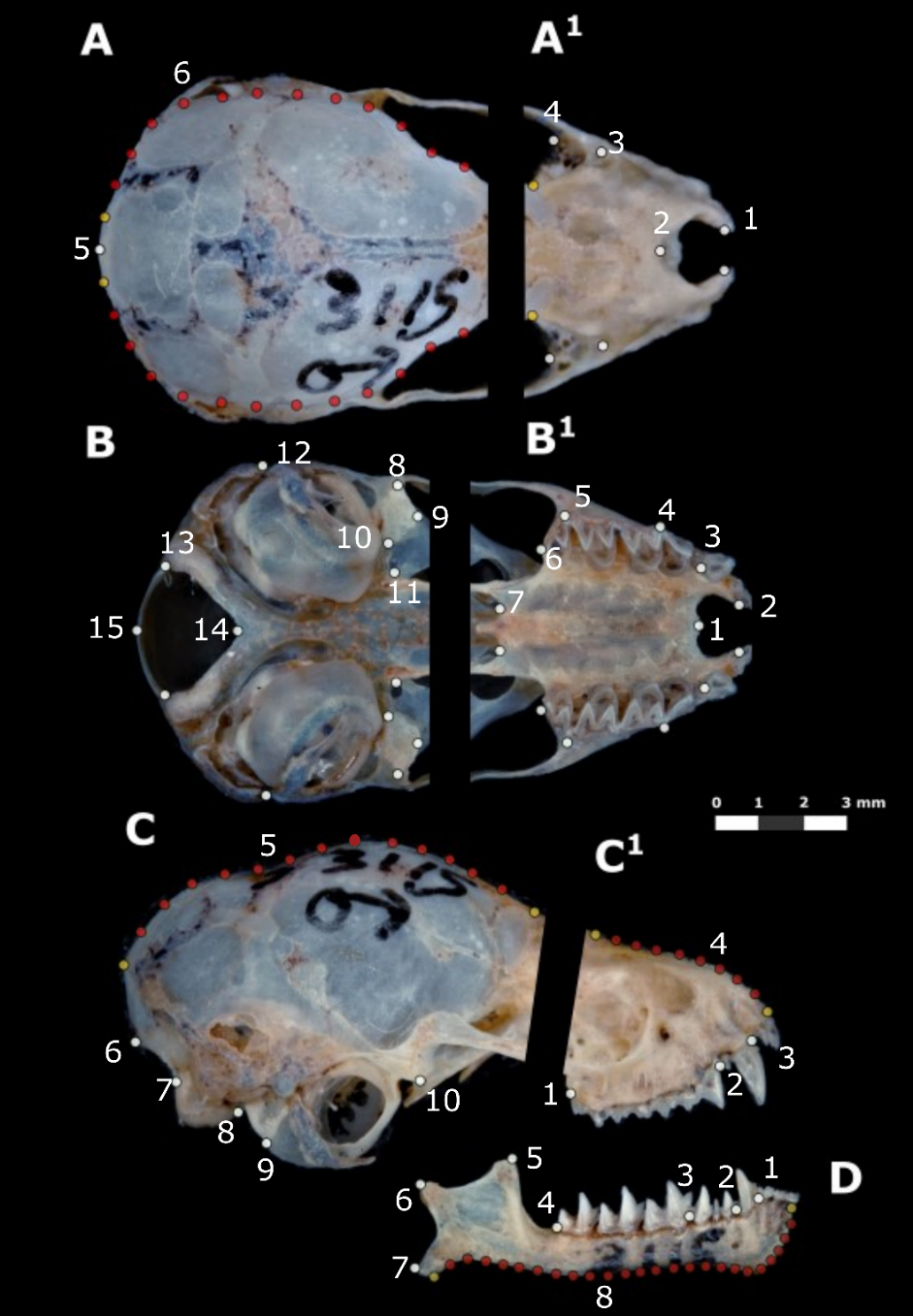


**Dorsal view of skull (A and A1)**

***Rostrum (A1)***

1. Distal edge of the alveolus of the first incisor.

2. Most proximal point of the internasal suture.

3. Most external point in the medial-lateral zone of the nasal bone.

4. Maximum curvature of the maxillary bone in the ocular area.

***Basicranium (A)***

5. Outermost point of the supraoccipital bone.

6. -14 semilandmarks: edge of the supraoccipital, parietal and frontal bones to their narrowest part.

**Ventral view of the skull (B and B1)**

***Rostrum (B)***

1. Most distal point of the intermaxillary suture.

2. Most distal edge of the alveolus of the first incisor.

3. Most proximal edge of the canine alveolus.

4. Metastyle of the first molar.

5. Metastyle of the third molar.

6. Paraconus of the third molar.

7. Maximum curvature of the palatine bone at the base of the postpalatal process.

***Basicranium (B1)***

8. Outermost border of the glenoid fossa.

9. Most distal border of the glenoid fossa.

10. Innermost border of the glenoid fossa.

11. Foramen oval of the sphenoid bone.

12. Most distal border of the squamous bone.

13. Most proximal border of the occipital condyle.

14. Most distal edge of the foramen magnum located at the base of the basioccipital.

15. Maximum curvature of the supraoccipital bone.

**Lateral view of the skull (C and C1)**

***Rostrum (C)***

1. Mesial edge of the alveolus of the third molar.

2. Distal border of the alveolus of the second premolar.

3. Distal edge of the canine alveolus.

4. -10 semi-landmarks: external border of the maxillary, nasal and premaxillary bones. From the front of maxillary suture to the distal edge of the alveolus of the first incisor.

***Basicranium (C1)***

5. -15 semi-landmarks on the outer border of the frontal and parental bones. From the front of manxillary suture to the interparietal suture.

6. Outermost point of the occipital-interparietal suture.

7. Upper border of the occipital condyle.

8. Lower border of the occipital condyle.

9. Suture of the cochlear promontory.

10. Outermost point of the glenoid fossa.

Jaw (D)

1. Most distal edge of the canine alveolus.

2. Most distal edge of the alveolus of first premolar.

3. Most distal edge of the alveolus of first molar.

4. Most mesial edge of the alveolus of third premolar.

5. Maximum curvature of the condylar process.

6. Maximum curvature of the coronoid process.

7. Maximum curvature of the condylar process.

8. Maximum curvature in the proximal region of the angular process.

9. -25 semi-landmarks on the outer edge of the mandible. From the maximum curvature in the distal region of the angular process to the most distal edge of the alveolus of the first incisor.

| **Table A.** Values of the sample size (n), mean, and standard deviation (SD) of the external morphological variables of *C. mexicanus* grouped by sex. The results of the *t* test and Mann-Whitney U test (*) and the value of the Cohen's D index (D) are shown. Mean and standard deviation are given in millimeters (mm). | | | | | | | |
| --- | --- | --- | --- | --- | --- | --- | --- |
|  | **Males** | | **Females** | | ***t*** | ***p*** | ***D*** |
|  | ***n*** | ***Mean ± SD*** | ***n*** | ***Mean ± SD*** |  |  |  |
| *Forearm* | 74 | 40.96 ± 0.99 | 77 | 42.23 ± 1.14 | 7.38 | < 0.001 | 1.2 |
| *Tibia* | 53 | 19.37 ± 0.97 | 60 | 19.83 ± 0.82 | 2.7 | 0.008 | 0.5 |
| *Ear* | 45 | 28.14 ± 1.21 | 63 | 28.65 ± 1.33 | 2.08 | 0.04 | 0.4 |
| *Tragus* | 53 | 10.82 ± 0.96 | 65 | 10.9 ± 0.82 | 0.49 | 0.62 | - |
|  | | | | | | | |
|  |  |  |  |  | ***w*** | ***p*** |  |
| *Number of interfemoral ridges** | 64 | 15.7 ± 1.91 | 72 | 16.3 ± 1.48 | 2717 | 0.07 | - |

| **Table B.** Values of the sample size (n), mean, and standard deviation (SD) of the external morphological variables of *C. mexicanus* grouped by sex and lineage. The results of the One-way ANOVA are shown. Mean and standard deviation are given in millimeters (mm). Abbreviation: d. f., degree of freedom. | | | | | | | | | | | | | |
| --- | --- | --- | --- | --- | --- | --- | --- | --- | --- | --- | --- | --- | --- |
|  |  | **SMO** | | | **SMOC** | | | **TMVB** | | |  |  |  |
|  | **sex** | **n** | **mean** | **SD** | **n** | **mean** | **SD** | **n** | **mean** | **SD** | **d. f.** | **F** | **p-value** |
| Forearm | Males | 7 | 40.44 | 0.92 | 19 | 40.52 | 0.65 | 48 | 41.20 | 1.03 | 2,71 | 4.73 | 0.012 |
| Tibia |  | 4 | 28.29 | 1.33 | 18 | 28.47 | 1.35 | 31 | 27.82 | 1.01 | 2,50 | 0.43 | 0.654 |
| Ear |  | 8 | 19.43 | 0.54 | 16 | 19.23 | 0.65 | 21 | 19.45 | 0.93 | 2,42 | 1.43 | 0.250 |
| Tragus |  | 7 | 10.26 | 0.73 | 17 | 11.09 | 0.88 | 29 | 10.79 | 1.01 | 2,50 | 1.96 | 0.152 |
| Forearm | Females | 14 | 42.04 | 1.38 | 32 | 42.53 | 1.03 | 31 | 42.19 | 1.14 | 2,74 | 0.40 | 0.668 |
| Tibia |  | 10 | 20.18 | 0.87 | 32 | 19.78 | 0.97 | 18 | 19.39 | 0.89 | 2,57 | 3.04 | 0.056 |
| Ear |  | 12 | 28.88 | 1.24 | 32 | 28.75 | 1.28 | 19 | 28.33 | 1.47 | 2,60 | 0.80 | 0.451 |
| Tragus |  | 10 | 10.88 | 0.93 | 30 | 11.04 | 0.74 | 25 | 10.74 | 0.87 | 2,62 | 0.95 | 0.392 |

| **Table C.** Values of the sample size (n), mean, and standard deviation (SD) of the external morphological variables of *C. mexicanus* grouped by sex. The results of the Kruska-Wallis test are shown. Mean and standard deviation are given in mm. Abbreviations: d. f., degree of freedom; IQR, interquartile range. | | | | | | | | | | | | | |
| --- | --- | --- | --- | --- | --- | --- | --- | --- | --- | --- | --- | --- | --- |
|  |  | **SMO** | | | **SMOC** | | | **TMVB** | | |  |  |  |
|  | **sex** | **n** | **median** | **IQR** | **n** | **median** | **IQR** | **n** | **median** | **IQR** | **d. f.** | **chi-square** | **p-value** |
| Number of interfemoral ridges | Males | 8 | 16 | 14-17 | 18 | 16 | 15-16.75 | 38 | 16 | 14.25-17 | 2 | 2.634 | 0.268 |
|  | Females | 13 | 16 | 15-17 | 32 | 16.5 | 16-18 | 27 | 16 | 15-17 | 2 | 5.560 | 0.062 |

| **Table D.** Summary of expected and predicted counts and percentages of reassignment of individuals to the original groups (cross-validation) according to Canonical Variate Analysis (CVA) results. Only modules with statistical significance shape variation are shown followed by the sample size (n). The confusion matrices indicate the expected group (in bold) and the predicted group (underline). The values in the confusion matrices correspond to the number of individuals (top) and the percentage with respect to the sample number (bottom). Abbreviations: SMO, Sierra Madre Oriental; SMOC, Sierra Madre Occidental, and TMVB, Trans-Mexican Volcanic Belt. | | | | | |
| --- | --- | --- | --- | --- | --- |
| *Dorsal rostrum Females* | n |  | TMVB | SMOC | SMO |
|  | 33 | **TMVB** | 22 | 9 | 2 |
|  |  |  | 66.60% | 27.20% | 6% |
|  | 26 | **SMOC** | 9 | 16 | 1 |
|  |  |  | 34.60% | 61.50% | 3.80% |
|  | 10 | **SMO** | 4 | 4 | 2 |
|  |  |  | 40% | 40% | 20% |
| *Lateral basicranium Males* | n |  |  |  |  |
|  | 34 | **TMVB** | 30 | 2 | 2 |
|  |  |  | 88.20% | 5.80% | 5.80% |
|  | 14 | **SMOC** | 3 | 9 | 2 |
|  |  |  | 21.40% | 64.20% | 14.20% |
|  | 7 | **SMO** | 3 | 0 | 4 |
|  |  |  | 42.80% | 0% | 57.10% |
| *Lateral basicranium Females* | n |  |  |  |  |
|  | 33 | **TMVB** | 24 | 8 | 1 |
|  |  |  | 72.70% | 24.20% | 3% |
|  | 27 | **SMOC** | 5 | 19 | 3 |
|  |  |  | 18.50% | 70.30% | 11.10% |
|  | 10 | **SMO** | 5 | 4 | 1 |
|  |  |  | 50% | 40% | 10% |
| *Mandible*  *Males* | n |  |  |  |  |
|  | 32 | **TMVB** | 25 | 5 | 2 |
|  |  |  | 78.10% | 15.60% | 6.20% |
|  | 14 | **SMOC** | 10 | 3 | 1 |
|  |  |  | 71.40% | 21.40% | 7.10% |
|  | 7 | **SMO** | 4 | 0 | 3 |
|  |  |  | 57.10% | 0% | 42.80% |
| *Mandible*  *Females* | n |  |  |  |  |
|  | 29 | **TMVB** | 14 | 15 | 0 |
|  |  |  | 48.20% | 51.70% | 0% |
|  | 25 | **SMOC** | 13 | 10 | 2 |
|  |  |  | 52% | 40% | 8% |
|  | 10 | **SMO** | 2 | 2 | 6 |
|  |  |  | 20% | 20% | 60% |
